# Correcting for discounting and loss aversion in composite time trade‐off

**DOI:** 10.1002/hec.4529

**Published:** 2022-04-26

**Authors:** Stefan A. Lipman, Arthur E. Attema, Matthijs M. Versteegh

**Affiliations:** ^1^ Erasmus Centre for Health Economics Rotterdam Erasmus School of Health Policy & Management Erasmus University Rotterdam Rotterdam Netherlands; ^2^ Institute for Medical Technology Assessment Erasmus University Rotterdam Rotterdam Netherlands

**Keywords:** discounting, loss aversion, reference‐dependence, time trade‐off

## Abstract

Time trade‐off utilities have been suggested to be biased upwards. This bias is a result of the method being applied assuming linear utility of life duration, which is violated when individuals discount future life years or are loss averse for health. Applying a “corrective approach”, that is, measuring individuals' discount function and loss aversion and correcting time trade‐off utilities for these individual characteristics, may reduce this bias in utilities. Earlier work has developed this approach for time trade‐off in a student sample. In this study, the corrective approach was extended to composite time trade‐off (cTTO) methodology, which enabled correcting utilities for health states worse than dead. In digital interviews a sample of 150 members of the general public completed cTTO tasks for six health states, and afterward they completed measurements of loss aversion and discounting. cTTO utilities were corrected using these measurements under multiple specifications. Respondents were also asked to reflect on and adjust their cTTO utilities directly. Our results show considerable loss aversion and both positive and negative discounting were prevalent. As predicted, correction generally resulted in lower utilities. This was in accordance with the direction of adjustments made by respondents themselves.

## 1 INTRODUCTION

Allocation of scarce health care resources can be informed by economic evaluation, in which costs associated with treatments are compared to the outcomes they yield (Drummond et al., 2015). These outcomes are often expressed as quality‐adjusted life‐years (QALYs), a measure of health comprising length of life and health‐related quality of life (HRQOL). Calculating QALYs requires a weight that represents the value or utility of a state of health, which is multiplied by the duration for which this state is experienced. A health state in which HRQOL is impaired, that is considered better than dead (BTD), receives a utility between 0 and 1, with negative utilities assigned to health states that are considered worse than dead (WTD).

The utilities required to calculate QALYs can be obtained by means of several health state valuation methods. Of these methods, the time trade‐off (TTO) remains particularly relevant, as it is used in valuation of EQ‐5D instruments (Ramos‐Goñi et al., 2020; Stolk et al., 2019), which are recommended for the measurement and valuation of HRQOL in countries such as the UK (NICE, 2018) and the Netherlands (ZINL, 2015). In TTO tasks, individuals are asked to imagine a life in impaired health, for example, 10 years in a wheelchair, for which a life time equivalent in perfect health is elicited, for example, 8 years in perfect health. The task is often (e.g., in EQ‐5D valuation) framed by asking respondents how much time in impaired health they would give up.

TTO is typically applied assuming the linear QALY model holds (Pliskin et al., 1980, defined in Section 2). This model assumes utility of life duration is linear, that is, future life years are not discounted. In practice, this assumption is violated for many individuals, who positively discount the future, which means they derive less utility from health in the future (Attema & Brouwer, 2014; Attema et al., 2012; Van Der Pol & Roux, 2005). On the other hand, negative discounting has been observed as well, that is, individuals assigning more weight to health in the future (e.g., Lipman & Attema, 2020; Van Der Pol & Cairns, 2000). Since the linear QALY model assumes no discounting, systematic deviations from this assumption could yield bias.

Another violation of the linear QALY model that may affect TTO is reference‐dependence (Kahneman & Tversky, 1979; Tversky & Kahneman, 1992), which entails that health outcomes are evaluated relative to a reference‐point. Outcomes considered better than the reference‐point are coined gains, while outcomes worse than the reference‐point are losses. This distinction is relevant when individuals are loss averse, that is, when losses loom larger than gains of the same size. Although loss aversion with respect to a reference‐point was established for monetary decision‐making, it has been found to apply to health outcomes as well (Kemel & Paraschiv, 2018; Lipman et al., 2019a). Loss aversion has been argued to lead to bias in TTO (Bleichrodt, 2002; Lipman et al., 2019c), assuming that the time spent in impaired health serves as reference‐point. An individual's expected life duration has also been suggested to serve as reference‐point (Lipman et al., 2020b; Van Nooten & Brouwer, 2004), with other authors suggesting reference‐points in the domain of HRQOL to be relevant in health contexts (Wouters et al., 2015).

If bias in TTO related to discounting and loss aversion is considered undesirable, earlier work suggests it may be corrected for (Attema & Brouwer, 2009; Lipman et al., 2019c; van Osch et al., 2004). Such a correction process typically involves approximating the degree of discounting and loss aversion and taking this into account when deriving TTO utilities (Lipman et al., 2019b). Several authors have explored correcting TTO for discounting (Attema & Brouwer, 2009; Attema et al., 2013; Van Der Pol & Roux, 2005; van Osch et al., 2004), but so far only one study applied such a corrective approach to TTO for both loss aversion and discounting (Lipman et al., 2019c). This study measured discounting and loss aversion using the non‐parametric method, developed by Abdellaoui et al. (2016), for each individual. TTO utilities were significantly lower after bias was corrected for, which is in accordance with earlier theoretical predictions (Bleichrodt, 2002) and empirical work (Lipman et al., 2020a).

However, several issues preclude the use of the corrective approach in practice (Lipman et al., 2019b). First, most work on correction for discounting and loss aversion is based on student samples in a lab‐setting, which hampers external validity. Second, most work on the corrective approach has focused on correcting TTO utilities for relatively mild health states. When severe health states are used, some respondents may provide responses suggesting they find health states WTD, which would require an alternative variant of TTO (Attema et al., 2013; Tilling et al., 2010) for which no corrective approach has yet been developed. Third, earlier studies have predominantly used self‐completed TTO, whereas interviewer‐assisted TTO data collection yields data of higher quality compared to (online) self‐completed TTO (Norman et al., 2010).

Hence, the main motivation of this study was to extend the approach developed by Lipman et al. (2019c) for use in valuation studies such as those for EQ‐5D (Ramos‐Goñi et al., 2020; Stolk et al., 2019). This extension involved: i) the use of (methods suitable for) a non‐student sample, ii) developing corrections for composite TTO, which uses lead‐time TTO for eliciting utilities for WTD health states (Attema et al., 2013; Ramos‐Goñi et al., 2020; Stolk et al., 2019), and iii) using computer‐assisted personal interviewing (following the protocol developed by Stolk et al., 2019).

The remainder of this paper is structured as follows. Section 2 defines our notational conventions, while Section 3 presents the extensions applied to the corrective approach used. Next, the experiment used to test this extended corrective approach is reported in Section 4. Section 5 presents the results of this experiment, which are discussed in Section 6.

## 2 NOTATION AND PRELIMINARIES

Preference notation is as usual, that is, ≻,≽, and ∼ represent strict preference, weak preference and indifference, respectively. For chronic health states, we will denote health profiles as (Q,T), that is, health state Q with duration T with. We will also write $\left(Q_{x},T_{x};\;Q_{y},T_{x}+1:T_{y}\right)$ to express a health profile in which quality of life is equal to $Q_{x}$ in $\text{periods}\;1,\;2,\;\text{…}\;\text{to}\;T_{x},$ followed by $Q_{y}\;$ for period $T_{x}+1,\;\text{…},\;T_{y}$. Note that subscripts are added to T and Q, for example, $T_{x},\;T_{y},\;Q_{x}$ and $Q_{y}$, only when needed to clarify which duration T and state *Q* applies to which period or outcome, and otherwise we will just write T or Q. If health profiles involve perfect quality of life (i.e., no impairments), we will express this duration in full health as (FH,T). In the general QALY model, preferences for health profiles of the form (Q,T) are evaluated by a utility function U(⋅) which comprises the utility of length of life, modeled by L(⋅), and quality of life, modeled by H(⋅):

(1) U(Q,T)=H(Q)∗L(T).

Using this notation, TTO indifferences elicited with the usual gauge duration of 10 years (Ramos‐Goñi et al., 2020; Stolk et al., 2019), that is, of the form (FH,T)∼(Q,10) are evaluated by: H(FH)∗L(T)=H(Q)∗L(10). If, as is usual, we assume H(FH)=1, we can derive the utility of health state Q as:

(2) H(Q)=L(T)/L(10).

If utility of life duration is assumed to be linear, as in the linear QALY model, that is, L(T)=T, Equation (2) simplifies to:

(3) H(Q)=T/10.

This TTO approach is not valid for eliciting utility for health profiles considered WTD. Different methods exist for eliciting such utilities (Augustovski et al., 2013; Tilling et al., 2010). In the recent valuation protocols for EQ‐5D valuation studies, the lead‐time TTO is used for this purpose (Ramos‐Goñi et al., 2020; Stolk et al., 2019). Lead‐time TTO involves choices between two health profiles: full health for some duration (i.e., the lead time) followed by impaired health for some duration. Typically, the lead time duration and the time in impaired health are equal (they need not be, but we will assume they are for simplicity), and both are often 10 years in practice (Ramos‐Goñi et al., 2020; Stolk et al., 2019). The other health profile, as in “conventional” TTO tasks, involves full health for some duration, of which the duration is typically varied until indifference is obtained. Using our notational conventions, such lead time TTO indifferences can be expressed as: (FH,T)∼(FH,10;Q,11:20). Under the general QALY model, such indifferences can be evaluated as: H(FH)∗L(T)=H(FH)∗L(10)+H(Q)∗(L(20)−L(10)), which assuming H(FH)=1, yields the utility of health state Q as:

(4) $$H=\frac{L\left(T\right)-L\left(10\right)}{L\left(20\right)-L\left(10\right)}.$$

If utility of life duration is assumed to be linear (L(T)=T), this simplifies to:

(5) $$H\left(Q\right)=\frac{T-10}{10}.$$

Although lead‐time TTO can yield both positive and negative utilities, that is, is suitable for valuation of both health states BTD and WTD, in valuation studies for EQ‐5D it is solely used for WTD health states (Ramos‐Goñi et al., 2020; Stolk et al., 2019). Such use of TTO for BTD health states and lead‐time TTO for WTD health states is referred to as the “composite TTO” (cTTO). By definition, the use of cTTO implies that utilities are elicited onto a single scale by two distinct tasks, involving trade‐offs at different points in time. If an individual's utility function for life duration is non‐linear, whether a period of impaired health occurs earlier or later will affect utilities (Attema & Versteegh, 2013), meaning that the use of cTTO without applying a corrective approach could be, at least conceptually, seen as problematic.

## 3 CORRECTIVE APPROACH FOR CTTO

In this paper, we will use and extend the approach developed in Lipman et al. (2019c) to derive corrections for cTTO. In order to extend the corrective approach to composite TTO, the approach should be extended to lead‐time TTO for WTD health states. Seeing as this is the main contribution of this paper, this is elaborated on in some detail.

The model developed by Lipman et al. (2019c) extends the general QALY model to accommodate insights from prospect theory in three ways, here summarized shortly. First, the model incorporates a reference‐point ($Q_{r},T_{r})$. Importantly, the reference‐point can be different between tasks. Second, we modify the scale for utility function for analytical convenience. That is, we apply a different scaling to utility of life duration such that L(0)=0 and L(20)=1, i.e., the utility of 0 life years is set to 0, and the utility of living 20 years is set to 1. The advantage of this scaling is, compared to the scaling used in (Lipman et al., 2019c), is that the zero condition is still satisfied, that is, the product of L(0) and H(Q) will always be 0 irrespective of the quality of life, reflecting the intuition that all health states are valued equally in the case of zero life duration (Miyamoto et al., 1998). In order to distinguish between gains and losses w.r.t. The reference‐point, we will rewrite the formula of the general QALY model Equation (1), to define evaluation of health profiles with respect to a reference‐point $\left(Q_{r},T_{r}\right)$ as follows:

(6) $$U\left(Q_{x},T_{x}\right)=H\left(Q_{r}\right)*L\left(T_{r}\right)+\left(H\left(Q_{x}\right)-H\left(Q_{r}\right)\right)*L\left(T_{x}\right)+H\left(Q_{r}\right)*\left(L\left(T_{x}\right)-L\left(T_{r}\right)\right).$$

In this expression, we decomposed the total utility into the utility of the reference‐point, a gain/loss part with respect to $Q_{r}$, and a gain/loss part with respect to $T_{r}$, respectively. Note that this decomposition is a modified expression of the general QALY model. This means that for any $Q_{x},T_{x},\;Q_{r}$ and $T_{r},\;$ the resulting utility derived through Equations (1) and Equation (6) are identical, as can be seen from Appendix A. Our addition to general QALY model is to introduce a loss aversion index λ to losses in *T*, that is, $T<T_{r}$, with λ>1(λ=1,λ<1) indicating loss aversion (loss neutrality, gain seeking). For gains in *T*, that is, $T\geq T_{r}$, as well as gains and losses in Q ($Q≽Q_{r}\;$ and $Q≺Q_{r}\;$ respectively), we assume no loss aversion (i.e., λ=1). Loss aversion is, thus, defined over life duration only, as it is not meaningful for health status, which is considered a qualitative measure. This model is a slightly modified version of the model proposed by Shalev (2002), that accounts for varying reference points, which we assume in this paper. If we multiply the loss in lifetime $L\left(T\right)-L\left(T_{r}\right)$ with λ, Equation (6) becomes:

$$U\left(Q_{x},T_{x}\right)=H\left(Q_{r}\right)*L\left(T_{r}\right)+\left(H\left(Q_{x}\right)-H\left(Q_{r}\right)\right)*L\left(T_{x}\right)+H\left(Q_{r}\right)*\lambda \left(L\left(T_{x}\right)-L\left(T_{r}\right)\right).$$

### 3.1 Corrective approach for TTO

As in earlier work (Bleichrodt, 2002; Lipman et al., 2019c) we will make the (simplifying) assumption that TTO indifferences of the form (FH,T)∼(Q,10) are elicited with (Q,10) as reference‐point (as this coincides with the framing typically used). That is, we have $Q_{r}=Q$ and $T_{r}=10$. According to Equation (6), this indifference can be evaluated by

(7) H(Q)L(10)+(H(FH)−H(Q))L(T)+H(Q)λ(L(T)−L(10)=H(Q)L(10).

From this expression, it becomes explicit that the option (FH,T) involves a gain in QoL, (H(FH)−H(Q))∗L(T), and a loss in lifetime spent in Q, H(Q)∗λ(L(T)−L(10)).

Given our scaling of H(FH)=1, solving for H(Q) yields:

(8) $$H\left(Q\right)=\frac{L\left(T\right)\;}{\lambda L\left(10\right)+\left(1-\lambda \right)L\left(T\right)}.$$

### 3.2 Corrective approach for lead‐time TTO

Applying a corrective approach to lead‐time TTO requires an assumption about the reference‐point in this method. Earlier qualitative work on gambles for length of life suggested that the outcome that remains constant across elicitations may serve as reference‐point (van Osch et al., 2006). In “conventional” TTO, this yielded the prediction that (Q,10) serves as reference‐point (Bleichrodt, 2002; Lipman et al., 2019c), for which some qualitative support can be found in Van Osch (2007). If this logic is symmetrically applied to lead‐time TTO, one could expect (FH,10;Q,11:20) to be the reference‐point. In cTTO, lead‐time TTO is only applied for WTD health states, which implies T<10. As such, if (FH,10;Q,11:20) is the reference‐point, then (FH,T) entails a loss of 10 years in Q and a loss of 10−T years in FH. Compared to the conventional TTO, we now have a reference health profile consisting of two instead of one chronic health states, but the same logic can be applied, see Appendix A. That is, (FH,T)∼(FH,10;Q,11:20), is evaluated as:

(9) H(FH)L(10)+H(Q)(L(20)−L(10))+H(FH)∗λ(L(T)−L(10))+H(Q)∗λ(L(10)−L(20))=H(FH)L(10)+H(Q)(L(20)−L(10)).

Solving for H(Q), and applying the scaling introduced earlier, gives:

(10) $$H\left(Q\right)=\frac{L\left(T\right)-L\left(10\right)}{1-L\left(10\right)}.$$

However, assuming (FH,10;Q,11:20) is taken as reference‐point, implies that we assume respondents take as reference‐point a health profile with a WTD health state and consider giving up life years in that state Q as a loss. This may be considered unlikely. Hence, we also apply our model assuming that respondents use a life duration of (FH,10) years as a reference‐point. In that case, respondents incur a loss in life duration (i.e., T−10 in FH) in the option (FH,T), and a gain in life time (i.e., 20−10) in Q in the option (FH,10;Q,11:20). The latter is in fact valued negatively because *Q* is a WTD health state. As such, (FH,T)∼(FH,10;Q,11:20) is evaluated by (see also Appendix A):

(11) H(FH)L(10)+H(FH)λ(L(T)−L(10))=H(FH)L(10)+H(Q)(L(20)−L(10)).

Solving for H(Q) and applying our scaling gives:

(12) $$H\left(Q\right)=\frac{\lambda \left(L\left(T\right)-L\left(10\right)\right)\;}{1-L\left(10\right)}.$$

Notice that, because in this case $T_{r}$  =  10, the only difference between Equations (10) and (12) is the addition of λ to the numerator of Equation (12). That is, H(Q) is predicted to be larger (i.e., less negative) if the reference point is (FH,10;Q,11:20) than if it is (FH,10) for λ>1.

## 4 EXPERIMENT

As demonstrated in the previous section, the corrective approach for cTTO can be operationalized either by correcting using Equations (8) and (10) or by Equations (8) and (12). The former approach, that is, based on Equations (8) and (10), assumes that respondents faced with TTO or lead‐time TTO use the constant outcome as reference‐point. This approach is referred to as *correction based on constant alternative* (in short: constant alternative correction). If on the other hand, we use the latter approach, that is, based on Equations (8) and (12), we assumed that the reference‐point is 10 years for both TTO and lead‐time TTO, which corresponds to the maximum time attainable in a BTD health state in both TTO and lead‐time TTO. As such, we refer to this approach as *correction based on maximum BTD time* (or in short: maximum BTD correction). Both these approaches involve different assumptions about the reference‐point for lead‐time TTO and no research is available to determine a priori which reference‐point individuals use. Therefore, both approaches were applied in our experiment in which 6 cTTO utilities were elicited as well as λ and the utility function L(T) on the domain 0–20.

### 4.1 Sample and data collection strategy

The sample for this experiment consisted of 150 respondents of the general public, recruited through a marketing company. The marketing company was instructed to recruit such that the sample was a reasonable reflection of the Dutch population in terms of age, gender and education level, but no strict quota were applied. We believe such non‐random sampling is warranted as this study aimed to extend and replicate findings of Lipman et al. (2019c) in the general public, rather than to obtain representative cTTO utilities. Respondents were recruited for taking part in an academic study on the value of health and were invited for personal interviews taking place on university campus. For completing the interview, which lasted around an hour, respondents were rewarded 30 euro. All interviews were completed in the Netherlands by the first author, using a personal laptop, in sessions of up to 7 interviews per day. Data collection commenced on March 8, 2020 and by March 13, 36 interviews were completed. The global outbreak of COVID‐19 and the lockdown of public facilities that followed it, however, necessitated a change in mode of administration, as face‐to‐face interviews were no longer possible. The remaining 114 interviews were completed digitally using videotelephony software (i.e., Zoom). The use of such software has several advantages and disadvantages for cTTO interviews, which are discussed elsewhere (Lipman, 2020). Table 1 shows respondent characteristics for the full sample, the sample that completed interviews in person, and the sample that completed interviews digitally. Furthermore, few differences existed between those sampled for personal or digital interviews, with only those sampled for digital interviews being slightly younger (*T*‐test, *p* = 0.001) and more likely to be married. We find no evidence of differences between the sample recruited for any of the other demographics reported in Table 1 (Chi‐squared tests, all *p*'s > 0.10).

**TABLE 1:** Demographics for the full sample and subsamples depending on data collection strategy

	Full sample (*n* = 150)	Personal interviews (*n* = 36)	Digital interviews (*n* = 114)
Sex			
Male	74 (49.3%)	13 (36.1%)	61 (53.5%)
Female	76 (50.7%)	23 (63.9%)	53 (46.5%)
Age (in years)			
Mean (SD)	42.7 (15.6)	50.3 (15.5)	40.3 (14.8)
Education level			
Low	10 (6.7%)	5 (13.9%)	5 (4.4%)
Middle	52 (34.7%)	11 (30.6%)	41 (36.0%)
High	88 (58.7%)	20 (55.6%)	68 (59.6%)
Household income			
<15,000 euros	40 (26.7%)	9 (25%)	31 (27.2%)
15,000–30,000 euros	44 (29.3%)	10 (27.8%)	34 (29.8%)
30,000–60,000s	42 (28%)	12 (33.3%)	30 (26.3%)
>60,000 euros	21 (14%)	3 (8.3%)	18 (15.8%)
Marital status			
Married	39 (26%)	15 (41.7%)	24 (21.1%)
Not married	111 (74%)	21 (58.3%)	90 (78.9%)
Current student			
Yes	12 (8%)	2 (5.6%)	10 (8.8%)
No	138 (92%)	34 (94.4%)	104 (91.2%)
Has children			
Yes	66 (44%)	20 (55.6%)	46 (40.4%)
No	84 (56%)	16 (44.4%)	68 (59.6%)
Religious			
Yes	33 (22%)	5 (13.9%)	28 (24.6%)
No	117 (78%)	31 (86.1%)	86 (75.4%)

*Note*: Education level was recoded as it was reported in terms of Dutch educational attainment. The following recoding is used: low education levels: VMBO, LBO or MAVO, middle education levels: VWO, MBO, or HAVO, and high education levels: HBO or WO.

### 4.2 Design

The interview protocol consisted of the following parts: a) Introduction and Demographics, b) cTTO introduction, c) main cTTO task for 6 states presented in randomized order (based on the EQ‐VT protocol), d) elicitation of loss aversion and discounting in randomized order, and e) a modification of the validation task developed by Lipman et al. (2020a). Ethical approval was provided by the Erasmus School of Health Policy's internal review board. Parts b) to c) were operationalized in Microsoft Powerpoint (using standardized EQ‐VT software), while d) and e) were operationalized in *R* Shiny. Each of these is elaborated on below (including how this was operationalized in digital interviews). The final design of this protocol was developed after conducting pilot sessions with 28 students and 5 test interviews with members of the general public. The main changes implemented after these pilot sessions involved clarifications of the instructions used and a reduction of the amount of health states in part c) from 10 to 6 to avoid fatigue in members of the general public.

#### 4.2.1 Introduction and Demographics

To commence the interview, the interviewer explained the goal of the interview (i.e., to measure the value of health in order to decide which treatment to fund), after which informed consent was obtained. In personal interviews written informed consent was provided, whereas in digital interviews informed consent was obtained and recorded verbatim. Afterward, a questionnaire was filled out capturing the following demographics (for details, see Appendix B): age, sex, income, subjective life expectancies (SLEs), religion, and beliefs about life after death and euthanasia (adapted from van Nooten et al., 2016). This part of the interview was concluded by respondents filling out the EQ‐5D‐5L instrument, that is, self‐reporting their health in terms of mobility, self‐care, ability to perform daily activities, pain or discomfort and anxiety or depression. Also, the EQ‐5D‐5L instrument contains a visual analog scale (EQ‐VAS) on which respondents report their health on a scale from 0 to 100, where 0 and 100 represent the worst and best imaginable health possible, respectively. In face‐to‐face interviews, respondents filled out the questionnaires on paper, in digital interviews respondents were shown the questionnaire and stated their answers verbatim which were stored by the interviewer.

#### 4.2.2 cTTO introduction and main cTTO task

Next, respondents were introduced to the cTTO task. The introduction used in this experiment is adapted from the EQ‐VT protocol, with slight modifications in place for our purposes. As is outlined in Stolk et al. (2019), cTTO was introduced to respondents by a “wheelchair” example, in which respondents are asked to imagine living for 10 more years in a wheelchair and are offered to live for 10 more years in perfect health instead. Next, the top‐down titration search procedure outlined in Oppe et al. (2014) was employed to elicit a cTTO indifference for this respondent. In both face‐to‐face and digital interviews, the respondent indicated their preference verbatim, which was entered into the software by the interviewer (for screenshots, see Appendix C). Next, respondents completed a second example employed to show the lead‐time component of cTTO (or equivalently the “conventional” TTO if life in a wheelchair was considered WTD).

All cTTO tasks were completed with health states described by EQ‐5D‐5L, that is, the EQ‐5D instrument that distinguishes five levels of severity on each of 5 domains of health‐related of life. This instrument uses the following five domains to described health‐related quality of life: mobility, self‐care, usual activities, pain/discomfort, and anxiety/depression, and describes problems on these domains with severity labels ranging from “no problems” to “extreme problems/unable to”. Health states are typically denoted by 5‐digit codes like 22113, with each number representing severity of the relevant domain. Respondents completed two practice cTTO tasks involving a relatively mild and severe health state (21211 and 35554 respectively). Next, for the main cTTO task, respondents completed a series of 6 cTTO tasks in succession for the following 6 health states (presented in random order): 11211, 13313, 35332, 22434, 24443, and 55555. These health states were selected to cover a range of health problems, from relatively mild to very severe and were also included in the Dutch valuation of EQ‐5D‐5L (Versteegh et al., 2016).

#### 4.2.3 Elicitation of loss aversion and discounting

Loss aversion was measured by means of the non‐parametric method (Abdellaoui et al., 2016). Note that this method can be used to measure the full prospect theory functional; that is, the utility for gains, utility for losses, probability weighting for gains and losses, and the loss aversion index. However, since we only need the loss aversion coefficient for our purposes, we only use the parts of this methodology required to assess loss aversion. This involves eliciting three chained indifferences (see Table 2 for an example), which allow estimating loss aversion as defined by Köbberling and Wakker (2005). The provision of an elaborate formal rationale for this method is beyond the scope of this paper, but they can be found in Abdellaoui et al. (2016) or the Online Supplements of Lipman et al. (2019c). Implementing this method for measuring loss aversion requires a reference‐point (denoted r) to which gains and losses are compared and a starting gain amount G from which the chained elicitation is started. To test the robustness of our corrective approach to different reference‐points, we measured loss aversion for two reference‐points, living for 10 and 20 more years.^1^ These years were described as being lived without health problems, as Lipman et al. (2019a) have shown the loss aversion coefficient estimated with this method does not depend systematically on the quality of life of the life duration gained and lost). Outcomes in the task were denoted as compared to this reference‐point (that is, +2 and −2 years denoted living for 12 and 8 years, respectively, when r=10). The gauge outcome G was set to 5 years.

**TABLE 2:** Indifferences elicited in the non‐parametric method, where $x_{0.5}y$ denotes a gamble yielding x with probability 0.5 and y otherwise and the example indifferences yield a loss aversion coefficient of λ=2

	General notation	Goal	Example
Indifference 1: Mixed prospect	G_0.5_ L ∼ r	Eliciting L	5_0.5_ −3∼0
Indifference 2: Certainty equivalence – gains	G_0.5_ r ∼ $x_{1}^{+}$	Eliciting $x_{1}^{+}$	5_0.5_ 0 ∼2
Indifference 3: Certainty equivalence ‐ losses	L_0.5_ r ∼ $x_{1}^{-}$	Eliciting $x_{1}^{-}$	−3_0.5_ 0 ∼−1
Köbberling and Wakker (2005)	$\lambda =\frac{x_{1}^{+}}{-x_{1}^{-}}$	Loss aversion coefficient	$\lambda =\frac{2}{-\left(-1\right)}=2$

Discounting (i.e., the curvature of the utility function L(T)) was elicited by means of the direct method (Attema et al., 2012). This method lets a subject compare two simple health profiles with the same time horizon, which are both combinations of two health states, for example, full health (FH) and some imperfect state Q that was operationalized by describing a state labeled chronic back pain (BP). This state was also described using EQ‐5D‐5L, that is, it was described as 21211. Both profiles had a 20‐year duration, which is assumed to be the reference‐point. Assuming our model holds, the use of the direct method provides the utility curvature of L(T) from L (0) to L(20). The difference between the profiles is that one starts with the better health state FH for some duration^2^ (denoted $T_{d1/2}$) and ends with the worse state BP for the remainder of the 20 years period (i.e., from $T_{d1/2}$ to 20). Using our notation this can be expressed as $\left(\text{FH},\;T_{d1/2};\text{BP},T_{d1/2}+1\;:20\right)$. The other health profile starts with BP for duration, followed by an improvement toward FH: that is, $\left(\text{BP},\;T_{d1/2};\;\text{FH},T_{d1/2}+1:20\right)$ Now, the purpose is to elicit the point $T_{d1/2}$ such that an individual is indifferent between the two profiles; that is, $\left(\text{FH},T_{d1/2};\;\text{BP},T_{d1/2}+1\;:20\right)∼\left(\text{BP},T_{d1/2};\;\text{FH},T_{d1/2}+1\;:20\right).$ Using our model, which does not involve losses in life duration and, hence, can be evaluated using the general QALY model, this indifference yields: $L\left(20\right)-L\left(T_{d1/2}\right)=L\left(T_{d1/2}\right)-L(0).$ Hence, the period $\left[1,T_{d1/2}\right]\;$ has the same utility as $\left[T_{d1/2},20\right]$. Given our normalization, *L* (0)=0andL(20)=1, this gives $L\left(T_{d1/2}\right)=1/2$ * [L(20)+L(0)]=1/2*1= 1/2. As is demonstrated in Attema et al. (2012), this procedure can be repeated by finding $T_{d1/4}\;$ such that L ($T_{d1/4})=$ $L\left(T_{d1/2}\right)-L\left(T_{d1/4}\right)$ and, hence, $L\left(T_{d1/4}\right)=1/4$. As a result, this method allows for a measurement of the utility function for life duration up to any desired precision. In this experiment, this procedure was performed 5 times, i.e., to determine the points T that yield $L(T_{d1/8})=\frac{1}{8},\;L(T_{d1/4})=\frac{1}{4},L(T_{d1/2})=\frac{1}{2},L(T_{d3/4})=\frac{3}{4},L(T_{d7/8})=\frac{7}{8}$. To apply Equations (8), (10) and (12) for any T, we use linear interpolation, which allows for correcting cTTO utilities without assuming a parametric form for L(T). The shape of L(T) can be characterized non‐parametrically by calculating the area under the curve (AUC). This AUC is calculated for a new function L*(*x*), where *x* = *T*/20, such that duration *T* is normalized to 0–1 scale. For this new function, the shape of L* (x) is concave [linear, convex] whenever AUC>0.5[AUC=0.5,AUC<0.5]. Although the corrective approach will be applied non‐parametrically, we also use the direct method data to estimate a discount rate with non‐linear least squares estimation and an exponential parametric form, that is: L* $\left(x\right)=\frac{1-\exp\left(-\rho x\right)}{1-\exp\left(-\rho \right)},$ which, as in our notation, yields L(0)=0 and L(T=20,x=1)=1. For ρ=0 we take L* (x)=x.

#### 4.2.4 Validation task

The final task performed in this experiment was an adaptation of the validation task developed by Lipman et al. (2020a). As in the original method, respondents are first explained the goal of QALYs and the role cTTO utilities play in calculating them (see Appendix C for screenshots of the task and the instruction used). Seeing the importance these utilities play in guiding allocation decisions, respondents are asked to reflect on what their choices imply about their views about the health states and their position on the QALY scale (i.e., from −1 to 1). This reflection has the following form. First, respondents are shown the utility elicited for a health state based on their stated preferences (based on Equations (3) and (5)) and asked to indicate if it: a) is exactly right, b) should be higher, c) should be lower. Afterward, respondents have the opportunity to specify a different utility for that health state with a slider between −1 and 1 using 2 decimals. Note that this validation task does not involve choice‐based trade‐offs of length and quality of life, but rather respondents reflect on, adjust (if necessary) and confirm utilities obtained for elicited stated preferences. The utilities derived from the cTTO task (obtained through Equations (3) and (5)) will be referred to as “elicited cTTO utilities” and the utilities confirmed by respondents are referred to as “confirmed cTTO utilities”.

### 4.3 Data analysis

Throughout we use a significance level of α=0.05. Seeing as many tests are reported, adjusting for multiple comparisons may be needed. Although many approaches for adjusting for multiple comparisons are defensible, in our analyses, Bonferroni adjusted *p*‐values are also reported whenever a single test is repeated multiple times. For example, when cTTO utilities before and after correction are compared for all 6 states with paired *t*‐tests, *p* values are multiplied by the number of tests (in this case 6). In such cases, *p* values are referred to as adjusted p's. Note that this approach is only applied to significant results, as Bonferroni adjustment is used to reduce the risk of Type I errors. Before further elaborating on data analysis, we compared data quality between digital and personal interviews, as differences between their data would warrant separate analysis and reporting of all results, or perhaps even exclusion of part of the sample. Next, we provided descriptive statistics for our sample, which also included loss aversion and discounting. Utilities are reported descriptively first, and a set of paired comparisons is used to compare confirmed, elicited and compared utilities per health state. Furthermore, the direction of correction was compared to the direction in which respondents adjusted their utilities themselves in the validation task.

## 5 RESULTS

### 5.1 Data quality

#### 5.1.1 Interviews completed

Out of the 150 interviews performed, 2 interviews were terminated before data could be collected for discounting and loss aversion, because it took over 50 min to complete parts a) to c) of the interview (i.e., measurement of cTTO utilities). To avoid having to cancel other interviews scheduled that day, these two interviews (1 personal and 1 digital interview) were ended prematurely. Furthermore, a single digital interview was terminated after approximately 20 min, after part b), as the respondent indicated to find deciding about health and trading off life years to be unacceptable due to religious reasons. As such, we have complete data for 147 respondents, and partial data (cTTO utilities only) for 149 respondents.

#### 5.1.2 Comparing personal and digital interviews and overall data quality

As is also discussed elsewhere (Lipman, 2020), we found no differences between digitally and personally completed TTO interviews (see Appendix D) on any of the quality indicators included in our analysis. That is, we find digital and personal interviews to both have a similar amount of problematic responses, as defined by Alava et al. (2020). Furthermore, Appendix D also reports a series of analyses that indicate that no difference existed in cTTO utilities between digital and personal interviews. Hence, all further analyses (including further analysis of data quality) are reported for the combined sample. When exploring data quality in the full sample, a relatively large amount of non‐trading and all‐in‐trading responses were observed. That is, 134 (15%) and 118 (13%) out of the total 894 states valued (6 per respondent) received cTTO utilities of 1 and −1 respectively. Furthermore, 40 (27%) out of 149 respondents assigned at least 1 state the same as 55555. However, this relevantly high percentage appears to be inflated by non‐trading responses, as only 15 (10%) out of 149 respondents had such counterintuitive preferences when non‐trading responses were excluded.

### 5.2 Descriptive statistics

Table 3 contains descriptive statistics for the various measures completed in the interviews. Each is discussed separately below.

**TABLE 3:** Frequency table for EQ‐5D‐5L and descriptive statistics for remaining demographics, loss aversion and discounting measures

	Level 1	Level 2	Level 3	Level 4	Level 5
EQ‐5D‐5L: Mobility	126	19	5		
EQ‐5D‐5L: Self‐care	147	3			
EQ‐5D‐5L: Usual activities	118	24	6	2	
EQ‐5D‐5L: Pain/discomfort	82	47	19	2	
EQ‐5D‐5L: Anxiety/depression	107	34	7	2	
	**Mean**	**SD**	**Median**	**Q1**	**Q3**
EQ‐VAS	80.67	12.14	80	75	90
SLE	83.95	8.07	85	80	89.25
SLE‐max	93.28	9.9	93	87	100
λ with RP(10years)	3.51	5.49	2	1.29	3.42
λ with RP(20years)	3.48	6.02	1.88	1	3.12
$T_{d1/8}$	2.64	1.27	2.5	2	3
$T_{d1/4}$	5.03	1.88	5	4	6
$T_{d1/2}$	9.67	2.23	10	8.25	11
$T_{d3/4}$	14.35	2.15	15	13.5	15.5
$T_{d7/8}$	16.8	1.98	17.5	16.5	17.5
AUC	0.49	0.08	0.50	0.44	0.53
ρ	0.21	1.45	0	−0.34	0.72

Abbreviation: SLE, subjective life expectancy.

#### 5.2.1 EQ‐5D‐5L and demographic questionnaire

Respondents were generally healthy, the far majority reporting no problems on each separate dimension (84%, 98%, 79%, 55% and 71% respectively), and 39% of the sample reported no health problems at all (i.e., 11111). The three most occurring health profiles were: 11111, 11112, and 11121. If the Dutch tariff (Versteegh et al., 2016) is used to translate these EQ‐5D‐5L health states to utilities, we find a mean utility of 0.89 (SD = 0.12). If we compare subjective life expectancy (SLE) to individuals' age, we find that respondents' remaining SLE was 41.24 years (SD = 18.46).

#### 5.2.2 Loss aversion and discounting

When loss aversion was measured with 10 years as reference‐point, we found 82%, 14% and 4% of respondents to be loss averse, gain seeking or loss neutral, respectively. When the reference‐point was set to 20 years, the proportion of respondents being loss averse was slightly lower, with more respondents being loss neutral, that is, at 73% (loss averse), 13% (gain seeking) and 14% (loss neutral). Nonetheless, the mean estimate of λ was not significantly different between reference‐points (paired *t*‐test: *t* (146) = 0.07, *p* = 0.94). Indeed, 75% of respondents were classified the same regardless of the RP used for measuring λ. In particular, 66% of respondents were loss averse throughout. Although a Chi‐squared analysis suggested that classification was not independent of the RP used, that is, $\chi^{2}\left(4,\;n=147\right)=43.94,\;p<0.001$, it is good to point out that the 75% agreement observed is only slightly larger than the 60% agreement expected assuming independence. We found no differences in loss aversion (for either reference‐point) for sex, marital status, student status and parental status (*t*‐test, all *p*'s > 0.13), with one exception: non‐students had higher loss aversion parameters estimated with a 20 years RP (*t*‐test, *p* = 0.02, adjusted *p* = 0.21). ANOVA analyses suggested that loss aversion was similar across education and income levels (all *p*'s > 0.32). Furthermore, neither measure of loss aversion was associated with age or SLE (Spearman correlation, all *p*'s > 0.16). This lack of systematic association between loss aversion and demographics was substantiated with separate multivariate linear regressions for both λ measures as dependent variables and all demographics as predictors. For both measures, none of the demographics significantly predicted λ in this multivariate model (all *p*'s > 0.23). Note also that both λ measures were not correlated with ρ, that is, we find no evidence for correlations between loss aversion and discounting (Pearson *r*'s < 0.04, *p*'s > 0.63).

At the aggregate level, we find little evidence for discounting, as can be seen from Table 3. However, when we classify respondents using AUC, we find less evidence for linear utility. That is, the shape of L(T) was concave for 37%, linear for 13% and convex for 49% of the sample. Hence, it appears that large heterogeneity exists in individuals' discounting. We found no significant differences in the shape of L(T) for sex, marital status, student status and parental status (*t*‐test, all *p*'s > 0.06), but those reporting to be religious had more convex L(T), that is, assigning more weight to the future (*t*‐test, *p* = 0.02, adjusted *p* = 0.08). Utility curvature was not associated with age or SLE (Spearman correlation, *p*'s > 0.06), and no differences were observed for education and income level (ANOVA, *p*'s > 0.36). A multivariate linear regression with AUC as dependent and all demographics as predictor confirmed this finding for religion (*p* < 0.005). Furthermore, in a multivariate model AUC was associated with age and education level (*p*'s < 0.03), such that older individuals and individuals with a higher education level have more concave L(T), that is, assigning more weight to health in the present. None of the other demographics was a significant predictor of the shape of L(T) (all *p*'s > 0.06).

### 5.3 Elicited, confirmed and corrected cTTO utilities

**FIGURE 1:**
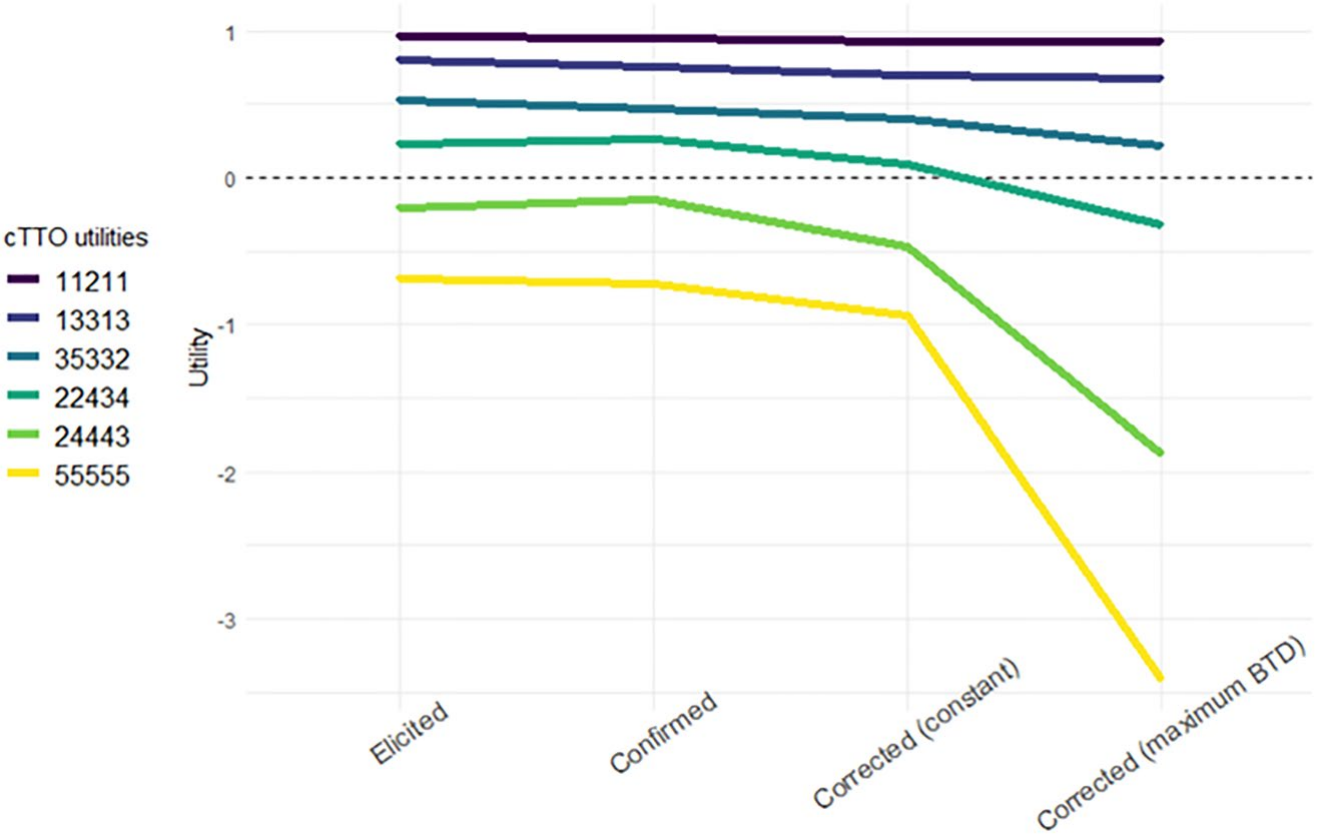
Mean utilities for all six health states elicited in this experiment

Table 4 reports the mean and median cTTO utilities elicited before and after correction (see also Figure 1), including the utilities “confirmed” by respondents in the validation task. Confirmed cTTO utilities were significantly lower than elicited cTTO utilities for all health states (paired *t*‐tests, all *p*'s < 0.03), except for state 24443 (paired *t*‐tests, *p* = 0.39). Depending on health state, 38%–69% left cTTO utilities unchanged in the validation task (i.e., equal to elicited utilities). The median number of changes each respondent made was 3, out of 6 health states. If a change was made, this was more likely to be downwards (21%–47% of the sample) than upwards (7%–29% of the sample) for all health states.

**TABLE 4:** Mean elicited, confirmed and corrected composite time trade‐off (cTTO) utilities, with standard deviations in brackets

State	11211	13313	35332	22434	24443	55555
Elicited	0.96 (0.07)	0.8 (0.24)	0.52 (0.48)	0.23 (0.59)	−0.21 (0.66)	−0.68 (0.42)
Confirmed	0.95 (0.07)	0.76 (0.21)	0.47 (0.4)	0.26 (0.46)	−0.15 (0.56)	−0.72 (0.4)
*Sig. (Elicited vs. confirmed)*	***	***	**		*^a^	*^a^
**Corrected**						
Constant alternative	0.93 (0.15)	0.7 (0.3)	0.4 (0.49)	0.09 (0.81)	−0.47 (1.38)	−0.94 (1.28)
Sig. (vs. Elicited)	***	***	*^a^	*^a^	**	***
Sig. (vs. Confirmed)	*^a^	*^a^		*^a^	**	***
Maximum BTD	0.93 (0.15)	0.67 (0.59)	0.22 (1.67)	−0.32 (3.3)	−1.88 (7.02)	−3.41 (7.79)
Sig. (vs. Elicited)	***	***	***	**	**	**
Sig. (vs. Confirmed)	*^a^	***	**	**	**	*^a^
**Differences**						
Elicited – confirmed utilities	0.02 (0.04)	0.04 (0.11)	0.06 (0.21)	−0.02 (0.28)	−0.05 (0.24)	0.04 (0.20)
Confirmed – corrected (constant alternative)	0.02 (0.12)	0.09 (0.44)	0.25 (1.55)	0.59 (3.13)	1.73 (6.86)	2.69 (7.77)
Sig (vs. Elicited‐confirmed)				*^a^	**	***
Confirmed – corrected (maximum BTD)	0.02 (0.12)	0.06 (0.17)	0.07 (0.30	0.16 (0.61)	0.31 (1.16)	0.22 (1.19)
Sig (vs. Elicited‐confirmed)				**	***	

^a^ indicates test was no longer significant after correcting for multiple testing.

*p < 0.05, **p < 0.01 and ***p < 0.001, indicate paired *t*‐test were significant with respectively.

Generally, corrected utilities were significantly lower than elicited utilities (paired *t*‐tests, all *p*'s < 0.03), and confirmed utilities (paired *t*‐tests, all *p*'s < 0.03). The only exception was state 35332, for which was not lower after constant alternative correction (paired *t*‐test, *p* = 0.053). Hence, although individuals adjusted their cTTO utilities downwards in the validation task, yielding lower confirmed than elicited utilities, corrected utilities were even lower. Note that these results are less pronounced when Bonferonni correction is applied (see Table 4). Interestingly, we find that for both corrective approaches for multiple states, the difference between elicited and confirmed cTTO utilities is smaller than the difference between confirmed and corrected cTTO utilities (paired *t*‐tests, all *p*'s < 0.01). These results that the corrective approach may be “overcorrecting”, which is an issue returned to in the Discussion.

Finally, we determined whether changes in confirmed utilities were in the direction predicted by the corrective approach. That is, we classified each upward or downward change in utilities as being “predicted” whenever it was in accordance with the direction of change implied by the corrective approach, and “unpredicted” if the corrective approach predicted no change or a change in the other direction. These findings can be found in Table 5. The majority of changes made by respondents was predicted by the corrective approach, which was a significant majority for 4 out of 6 health states (Chi‐squared tests, *p*'s < 0.004, adjusted *p*'s < 0.025), with health state 35332 and 55555 as exceptions (Chi‐squared tests, *p*'s > 0.06). As can be seen from Table 5, both corrective approaches yield the same qualitative results. Nonetheless, corrected utilities for constant correction were significantly lower for severe health states (in which lead‐time TTO was most likely to be encountered): that is, 24443 and 55555 (Wilcoxon tests, all *p*'s < 0.008, adjusted *p*'s < 0.05).

**TABLE 5:** Overview of number of respondents who made changes for confirmed utilities classified by predictions made by the corrective approach

	Constant correction					
**Health state**	**11211**	**13313**	**35332**	**22434**	**24443**	**55555**
Downward: Predicted	54	41	36	25	19	15
Downward: Unpredicted	3	20	35	29	12	17
Upward: Predicted	15	19	19	33	41	9
Upward: Unpredicted	1	1	2	2	3	2

	Maximum BTD correction					
**Health state**	**11211**	**13313**	**35332**	**22434**	**24443**	**55555**
Downward: Predicted	54	41	36	25	19	15
Downward: Unpredicted	3	20	35	29	12	17
Upward: Predicted	15	19	19	33	41	9
Upward: Unpredicted	1	1	2	2	3	2

## 6 DISCUSSION

With this project we aimed to extend the corrective approach for use in valuation studies such as those used for valuation of EQ‐5D, by developing corrections for cTTO. This paper has several strengths compared to earlier work applying a corrective approach. First, it is the first applying a corrective approach with interviewer‐assisted data collection with members of the general public. Some authors have explored correction for discounting in cTTO in the general public using online self‐completed data collection (Attema & Brouwer, 2014), but this mode of administration will generally lead to lower quality data and increased no‐shows (Norman et al., 2010). Furthermore, our results may be of larger practical relevance, as cTTO utilities were obtained following the EQ‐VT protocol. Data in this project was generally of high quality, even when the interviews were facilitated through videotelephony software (see Appendix D). Second, as parameters needed for applying a corrective approach were obtained at an individual level, this paper allows exploring heterogeneity in individuals' decision‐making in cTTO. The inclusion of the validation task developed by Lipman et al. (2020a) also enables exploring the validity of the corrective approach at the individual level.

Generally, the estimates of loss aversion and discounting are in accordance with earlier work. The median loss aversion estimate is close the initial estimate (i.e., 2.25) elicited for financial decision‐making in Kahneman and Tversky's (1979) work. Whereas earlier work has shown that loss aversion for life duration is independent of the quality of life described for its measurement (Lipman et al., 2019a), our paper adds to this literature that loss aversion is mostly unaffected by the reference‐point described (i.e., living for 10 or 20 more years). Our results for discounting suggest that the median discount function is linear, but we find large heterogeneity, suggesting that no single mean discount function can be applied to correct TTO responses and that it is sample‐specific, hence adding to the task burden of valuation studies. Such a linear curve for L(T) was also observed in Lipman et al. (2019a), but we find large heterogeneity. Many respondents have a concave shape for L(T), that is, reflecting positive discounting. Nonetheless, we observed negative discounting for the majority of respondents, which implies that discounting should be measured with methods flexible enough to capture both positive and negative discounting.

In this paper, we extended the corrective approach for lead‐time TTO, meaning that it can now be readily applied to correct cTTO utilities. We applied two different approaches, which differed in terms of the assumptions made about the reference‐point for lead‐time TTO. As a result, we find no difference between corrected cTTO utilities between the two approaches for relatively mild health states (as lead‐time TTO is unlikely to be required in these cases). The choice of reference‐point for the corrective approach, however, has a significant impact on corrected utilities for severe states. Future work should explore means of determining which reference‐point respondents used, for example, through decision process tracing (Pachur et al., 2018) or qualitative methods (van Osch et al., 2006). In line with our observation of no/little discounting on average, if we correct for loss aversion only, or discounting only (see Appendix D), we find that the downward trend observed when applying a corrective approach to cTTO utilities is exclusively driven by loss aversion (as in Lipman et al., 2019c). This finding is in contrast to earlier work using the direct method that has suggested that correcting for discounting would influence TTO utilities (Attema & Brouwer, 2014). Future work should aim to replicate our results, as considered in isolation they could suggest that, if only average utilities are of importance, correcting for discounting may not be necessary.

To our knowledge, this is only the second study to ask respondents to reflect on cTTO utilities on a cardinal scale. As in the first study (Lipman et al., 2020a), we find that cTTO utilities were more likely to be adjusted downwards than upwards. Hence, these findings also appear to apply to more severe health states. Although one may be inclined to interpret this as suggesting a corrective approach is needed, at least two caveats deserve mentioning. First, our findings suggest that in most cases cTTO utilities are left unchanged. This can be interpreted multiple ways. For example, respondents may have seen no need for adjusting the elicited utilities, but it could also be argued that respondents were confused by the task and left utilities unchanged for that reason. Second, confirmed utilities may have been lower due to respondents who feel that giving up life‐years is so undesirable that improved quality of life cannot easily offset it. In the validation task, no trade‐offs are required, and hence such non‐trading may be less pronounced. The corrective approach can capture such reluctance to trade‐off life years by incorporating loss aversion to a degree, but is not applicable to lexicographic non‐trading (i.e., loss aversion predicts life years are still given up albeit reluctantly).

Interestingly, corrected utilities were generally lower than confirmed utilities. How this discrepancy should be interpreted depends on which (if any) of the cTTO utilities reported in Table 4, one views as the best representation of individuals' judgments about the value of impaired relative to perfect health. Elicited cTTO utilities were highest and were derived with the state‐of‐the‐art approach used for health state valuation in practice (Stolk et al., 2019). However, one may feel that these utilities are unfit as benchmark, given that they are obtained while assuming no discounting or loss aversion. Both current literature and findings reported in this study provide ample challenge of these assumptions. It is not clear, on the other hand, if confirmed cTTO utilities provide a suitable benchmark to compare against. Confirmed cTTO utilities were obtained after respondents considered the goal of health state valuation and the scaling used for QALYs. Respondents that adjusted elicited utilities may have identified cases in which health states were assigned utilities that are too high or low, suggesting that corrected utilities are lower than necessary. The latter statement, would, however, appear to assign respondents significant introspective capability and sophistication, as it assumes they are able to identify most or all cases of biased elicited utilities and the method used for adjustment is not biased. Moreover, it is widely believed that preferences are shaped by the task with which they are elicited (Braga & Starmer, 2005), and hence any differences between confirmed and elicited utilities and cTTO utilities may merely be reflections of the different tasks used. Furthermore, it is well‐known that individuals may be “anchoring” on previous information (Tversky & Kahneman, 1974), in this case elicited utilities, and as a result adjust insufficiently. Thus, an argument may as well be made in favor of the lower corrected utilities to be used as benchmark, if one believes individuals' adjustments were only partial. Hence, given that it is debatable if true “utilities” exist or can be measured (Braga & Starmer, 2005), the interpretation of the utilities presented in this paper and the differences between them remains unclear. Additional work discussing the psychological realism and normative implications of the corrective approach appears warranted (e.g., Infante et al., 2016).

Nonetheless, three limitations of using the corrective approach developed in this paper should be mentioned. First, correcting cTTO utilities involves taking into account additional error in health state valuation. That is, measurement of time preference and loss aversion is subject to error, which may be especially true for chained methods such as the non‐parametric method (Abdellaoui et al., 2016) and the direct method (Attema et al., 2012). Although earlier work suggested there is little evidence for error propagation in such chained methods (Bleichrodt & Pinto, 2000; Lipman et al., 2019c), the two additional parameters required to correct elicited cTTO utilities may increase variability in utilities. However, when utilities are applied in practice, this is often based on the average of the point estimates as estimated from a tariff, disregarding the parameter uncertainty in the tariff itself (for a discussion, see: Devlin et al., 2017). Information about the variance in utilities is, thus, typically disregarded (for an exception, see: Versteegh et al., 2019). Second, the corrective approach implies that cTTO utilities are no longer bounded at −1, and as a result, utilities for WTD health states were much lower after correction. In this study, this may be problematic as the scale used to confirmed utilities was bounded at −1, which may also explain why confirmed utilities were higher than corrected utilities. The lack of a lower bound may be seen as problematic in practice (Tilling et al., 2010), but there is no normative basis for such a lower bound to exist. In fact, the cTTO approach applied in EQ‐VT arbitrarily sets this bound at −1 and if alternative approaches for valuation of WTD health states would have been incorporated, the lower bound would have been different (Attema et al., 2013; Attema & Versteegh, 2013; Augustovski et al., 2013). Third, in line with Lipman et al. (2019c) the corrective approach applied in this paper models loss aversion for life duration only. That is, life duration that exceeds some reference‐point is considered to be gained, whereas life duration that falls short of the reference‐point is considered lost. In this approach, the health state experienced in the life duration gained or lost does not impact loss aversion. This may have somewhat counterintuitive consequences, as for example, life years in a state WTD that exceed the reference‐point would be considered to be gained, whereas life years “given up” compared to a reference‐point in a state WTD are considered losses (and thus multiplied by a coefficient capturing loss aversion). This limitation may be addressed in future work expanding our approach to include loss aversion for Q, although this may be challenging given that Q is typically considered a qualitative measure for which loss aversion is undefined (Bleichrodt & Miyamoto, 2003).

To conclude, in this paper we have provided the foundations for the corrective approach to be used for health state valuation in practice. The methods used for measuring loss aversion and discounting were applied in a sample of the general public, and the corrective approach was extended to incorporate lead‐time TTO in cTTO. As in earlier work, correction has a downward effect on cTTO utilities for both mild and severe health states, which is largely driven by correction for loss aversion. The need to correct for loss aversion depends, however on which reference‐point is taken by respondents, and the required methods for enabling such correction have only recently been developed. Even though loss aversion appears a robust phenomenon in decisions about health, whether and how to account for its influence in health state valuation are still open questions.

## CONFLICT OF INTEREST

Matthijs Versteegh is a member of the EuroQol Group. All authors have received research grants from the EuroQol Research Foundation for work outside the scope of the submitted work.

## Supporting information

Supplementary Material

## Data Availability

The data that support the findings of this study are available from the corresponding author upon reasonable request.

## ENDNOTES

^1^
Note that the two reference‐points were included as we expected a priori that both could be relevant for correcting cTTO depending on the reference‐point assumed. However, in the final specification only λ elicited with a 10 year reference‐point is needed in Equations (8) and (12).

^2^

*T*_*d*1/2_ stands for the time point up until which half of the total utility of life duration is experienced, as explained in the remainder of this paragraph.

## References

[hec4529-bib-0001] Abdellaoui, M. , Bleichrodt, H. , L’haridon, O. , & Van Dolder, D. (2016). Measuring loss aversion under ambiguity: A method to make prospect theory completely observable. Journal of Risk and Uncertainty, 52, 1–20. 10.1007/s11166-016-9234-y

[hec4529-bib-0002] Alava, M. H. , Pudney, S. , & Wailoo, A. (2020). The EQ‐5D‐5L value set for England: Findings of a quality assurance program. Value in Health, 23(5), 642–648. 32389230 10.1016/j.jval.2019.10.017

[hec4529-bib-0003] Attema, A. E. , Bleichrodt, H. , & Wakker, P. P. (2012). A direct method for measuring discounting and QALYs more easily and reliably. Medical Decision Making, 32(4), 583–593. 10.1177/0272989x12451654 22706639

[hec4529-bib-0004] Attema, A. E. , & Brouwer, W. B. (2009). The correction of TTO‐scores for utility curvature using a risk‐free utility elicitation method. Journal of Health Economics, 28(1), 234–243. 10.1016/j.jhealeco.2008.10.004 19062114

[hec4529-bib-0005] Attema, A. E. , & Brouwer, W. B. (2014). Deriving time discounting correction factors for TTO tariffs. Health Economics, 23(4), 410–425. 10.1002/hec.2921 23564665

[hec4529-bib-0006] Attema, A. E. , & Versteegh, M. M. (2013). Would you rather be ill now, or later? Health Economics, 22(12), 1496–1506. 10.1002/hec.2894 23229912

[hec4529-bib-0007] Attema, A. E. , Versteegh, M. M. , Oppe, M. , Brouwer, W. B. , & Stolk, E. A. (2013). Lead time TTO: Leading to better health state valuations? Health Economics, 22(4), 376–392. 10.1002/hec.2804 22396243

[hec4529-bib-0008] Augustovski, F. , Rey‐Ares, L. , Irazola, V. , Oppe, M. , & Devlin, N. J. (2013). Lead versus lag‐time trade‐off variants: Does it make any difference? The European Journal of Health Economics, 14(3), 25–31. 10.1016/j.jval.2013.03.205 PMC3728455 23900662

[hec4529-bib-0009] Bleichrodt, H. (2002). A new explanation for the difference between time trade‐off utilities and standard gamble utilities. Health Economics, 11(5), 447–456. 10.1002/hec.688 12112493

[hec4529-bib-0010] Bleichrodt, H. , & Miyamoto, J. (2003). A characterization of quality‐adjusted life‐years under cumulative prospect theory. Mathematics of Operations Research, 28(1), 181–193. 10.1287/moor.28.1.181.14261

[hec4529-bib-0011] Bleichrodt, H. , & Pinto, J. L. (2000). A parameter‐free elicitation of the probability weighting function in medical decision analysis. Management Science, 46(11), 1485–1496. 10.1287/mnsc.46.11.1485.12086

[hec4529-bib-0012] Braga, J. , & Starmer, C. (2005). Preference anomalies, preference elicitation and the discovered preference hypothesis. Environmental and Resource Economics, 32(1), 55–89. 10.1007/s10640-005-6028-0

[hec4529-bib-0013] Devlin, N. , Shah, K. , & Buckingham, K. (2017). What is the normative basis for selecting the measure of “average” preferences for use in social choices. OHE research paper. Office of Health Economics. https://www.ohe.org/system/files/private/publications/OHE%20RP%20(Devlin%20et%20al.%20average%20preferences)%20FINAL.pdf

[hec4529-bib-0014] Drummond, M. F. , Sculpher, M. J. , Claxton, K. , Stoddart, G. L. , & Torrance, G. W. (2015). Methods for the economic evaluation of health care programmes. Oxford university press.

[hec4529-bib-0015] Infante, G. , Lecouteux, G. , & Sugden, R. (2016). Preference purification and the inner rational agent: A critique of the conventional wisdom of behavioural welfare economics. Journal of Economic Methodology, 23, 1–25. 10.1080/1350178x.2015.1070527

[hec4529-bib-0016] Kahneman, D. , & Tversky, A. (1979). Prospect theory: An analysis of decision under risk. Econometrica, 47(2), 263–291. 10.2307/1914185

[hec4529-bib-0017] Kemel, E. , & Paraschiv, C. (2018). Deciding about human lives: An experimental measure of risk attitudes under prospect theory. Social Choice and Welfare, 51(1), 163–192. 10.1007/s00355-018-1111-y

[hec4529-bib-0018] Köbberling, V. , & Wakker, P. P. (2005). An index of loss aversion. Journal of Economic Theory, 122(1), 119–131. 10.1016/j.jet.2004.03.009

[hec4529-bib-0019] Lipman, S. A. (2020). Time for tele‐TTO? Lessons learned from digital interviewer‐assisted time trade‐off data collection. The Patient, 14(5), 459–469. 10.1007/s40271-020-00490-z 33345290 PMC7750113

[hec4529-bib-0020] Lipman, S. A. , & Attema, A. E. (2020). Good things come to those who wait—decreasing impatience for health gains and losses. PLoS One, 15(3), e0229784. 10.1371/journal.pone.0229784 32126119 PMC7053719

[hec4529-bib-0021] Lipman, S. A. , Brouwer, W. B. , & Attema, A. E. (2019a). A QALY loss is a QALY loss is a QALY loss: A note on independence of loss aversion from health states. The European Journal of Health Economics, 20(3), 419–426. 10.1007/s10198-018-1008-9 PMC6438936 30229374

[hec4529-bib-0022] Lipman, S. A. , Brouwer, W. B. F. , & Attema, A. E. (2019b). The corrective approach: Policy implications of recent developments in QALY measurement based on prospect theory. Value in Health, 22(7), 816–821. 10.1016/j.jval.2019.01.013 31277829

[hec4529-bib-0023] Lipman, S. A. , Brouwer, W. B. F. , & Attema, A. E. (2019c). QALYs without bias? Non‐parametric correction of time trade‐off and standard gamble weights based on prospect theory. Health Economics, 28(7), 843–854. 10.1002/hec.3895 31237093 PMC6618285

[hec4529-bib-0025] Lipman, S. A. , Brouwer, W. B. , & Attema, A. E. (2020a). What’s it going to be, TTO or SG? A direct test of the validity of health state valuation. Health Economics, 29(11), 1475–1481. 32744408 10.1002/hec.4131 PMC7689723

[hec4529-bib-0024] Lipman, S. A. , Brouwer, W. B. F. , & Attema, A. E. (2020b). Living up to expectations: Experimental tests of subjective life expectancy as reference point in time trade‐off and standard gamble. Journal of Health Economics, 71, 102318. 10.1016/j.jhealeco.2020.102318 32229049

[hec4529-bib-0026] Miyamoto, J. M. , Wakker, P. P. , Bleichrodt, H. , & Peters, H. J. (1998). The zero‐condition: A simplifying assumption in QALY measurement and multiattribute utility. Management Science, 44(6), 839–849. 10.1287/mnsc.44.6.839

[hec4529-bib-0027] NICE . (2018). Guide to the processes of technology appraisal. In N. I. F. H. A. C. (Ed.), Excellence. https://www.nice.org.uk/Media/Default/About/what-we-do/NICE-guidance/NICE-technology-appraisals/technology-appraisal-processes-guide-apr-2018.pdf 27905710

[hec4529-bib-0028] Norman, R. , King, M. T. , Clarke, D. , Viney, R. , Cronin, P. , & Street, D. (2010). Does mode of administration matter? Comparison of online and face‐to‐face administration of a time trade‐off task. Quality of Life Research, 19(4), 499–508. 10.1007/s11136-010-9609-5 20174998

[hec4529-bib-0029] Oppe, M. , Devlin, N. J. , Van hout, B. , Krabbe, P. F. , & De Charro, F. (2014). A program of methodological research to arrive at the new international EQ‐5D‐5L valuation protocol. Value in Health, 17(4), 445–453. 10.1016/j.jval.2014.04.002 24969006

[hec4529-bib-0030] Pachur, T. , Schulte‐Mecklenbeck, M. , Murphy, R. O. , & Hertwig, R. (2018). Prospect theory reflects selective allocation of attention. Journal of Experimental Psychology: General, 147(2), 147–169. 10.1037/xge0000406 29369680

[hec4529-bib-0031] Pliskin, J. S. , Shepard, D. S. , & Weinstein, M. C. (1980). Utility functions for life years and health status. Operations Research, 28(1), 206–224. 10.1287/opre.28.1.206

[hec4529-bib-0032] Ramos‐goñi, J. M. , Oppe, M. , Stolk, E. , Shah, K. , Kreimeier, S. , Rivero‐Arias, O. , & Devlin, N. (2020). International valuation protocol for the EQ‐5D‐Y‐3L. PharmacoEconomics, 1–11. 10.1007/s40273-020-00909-3 32297224

[hec4529-bib-0033] Shalev, J. (2002). Loss aversion and bargaining. Theory and Decision, 52(3), 201–232. 10.1023/a:1019674323804

[hec4529-bib-0034] Stolk, E. , Ludwig, K. , Rand, K. , Van Hout, B. , & Ramos‐Goñi, J. M. (2019). Overview, update, and lessons learned from the international EQ‐5D‐5L valuation work: Version 2 of the EQ‐5D‐5L Valuation Protocol. Value in Health, 22(1), 23–30. 10.1016/j.jval.2018.05.010 30661630

[hec4529-bib-0035] Tilling, C. , Devlin, N. , Tsuchiya, A. , & Buckingham, K. (2010). Protocols for time tradeoff valuations of health states worse than dead: A literature review. Medical Decision Making, 30(5), 610–619. 10.1177/0272989x09357475 20068144

[hec4529-bib-0036] Tversky, A. , & Kahneman, D. (1974). Judgment under uncertainty: Heuristics and biases. Science, 185(4157), 1124–1131. 10.1126/science.185.4157.1124 17835457

[hec4529-bib-0037] Tversky, A. , & Kahneman, D. (1992). Advances in prospect theory: Cumulative representation of uncertainty. Journal of Risk and Uncertainty, 5(4), 297–323. 10.1007/bf00122574

[hec4529-bib-0038] Van Der Pol, M. , & Roux, L. (2005). Time preference bias in time trade‐off. The European Journal of Health Economics, 6(2), 107–111. 10.1007/s10198-004-0265-y 19787847

[hec4529-bib-0039] Van Der Pol, M. M. , & Cairns, J. A. (2000). Negative and zero time preference for health. Health Economics, 9(2), 171–175. 10.1002/(sici)1099-1050(200003)9:2<171::aid-hec492>3.0.co;2-z 10721018

[hec4529-bib-0040] Van Nooten, F. , & Brouwer, W. (2004). The influence of subjective expectations about length and quality of life on time trade‐off answers. Health Economics, 13(8), 819–823. 10.1002/hec.873 15322993

[hec4529-bib-0041] Van Nooten, F. , Van Exel, N. , Eriksson, D. , & Brouwer, W. (2016). Back to the future: Influence of beliefs regarding the future on TTO answers. Health and Quality of Life Outcomes, 14(1), 4. 10.1186/s12955-015-0402-6 26753687 PMC4709901

[hec4529-bib-0042] Van Osch, S. M. , Van Den Hout, W. B. , & Stiggelbout, A. M. (2006). Exploring the reference point in prospect theory: Gambles for length of life. Medical Decision Making, 26(4), 338–346. 10.1177/0272989x06290484 16855123

[hec4529-bib-0043] Van Osch, S. M. , Wakker, P. P. , Van Den Hout, W. B. , & Stiggelbout, A. M. (2004). Correcting biases in standard gamble and time tradeoff utilities. Medical Decision Making, 24(5), 511–517. 10.1177/0272989x04268955 15359000

[hec4529-bib-0044] Van Osch, S. M. C. (2007). The construction of health state utilities. Leiden University.

[hec4529-bib-0045] Versteegh, M. M. , Ramos, I. C. , Buyukkaramikli, N. C. , Ansaripour, A. , Reckers‐Droog, V. T. , & Brouwer, W. B. (2019). Severity‐adjusted probability of being cost effective. PharmacoEconomics, 37(9), 1155–1163. 10.1007/s40273-019-00810-8 31134467 PMC6830403

[hec4529-bib-0046] Versteegh, M. M. , Vermeulen, K. M. , M A A Evers, S. , Evers, S. M. , De Wit, G. A. , Prenger, R. , A Stolk, E. , & Stolk, E. A. (2016). Dutch tariff for the five‐level version of EQ‐5D. Value in Health, 19(4), 343–352. 10.1016/j.jval.2016.01.003 27325326

[hec4529-bib-0047] Wouters, S. , Van Exel, N. J. , Rohde, K. I. , & Brouwer, W. B. (2015). Are all health gains equally important? An exploration of acceptable health as a reference point in health care priority setting. Health and Quality of Life Outcomes, 13(1), 79. 10.1186/s12955-015-0277-6 26055258 PMC4460854

[hec4529-bib-0048] ZINL . (2015). Richtlijn voor het uitvoeren van economische evaluaties in de gezondheidszorg. Zorginstituut Nederland. https://www.zorginstituutnederland.nl/publicaties/publicatie/2016/02/29/richtlijn-voor-het-uitvoeren-van-economische-evaluaties-in-de-gezondheidszorg

