# Supplementary material for: Correcting for discounting and loss aversion in composite time trade‐off
Source: Health Econ. 2022 Apr 26;31(8):1633–48. doi: 10.1002/hec.4529 (PMC9541376; doi:10.1002/hec.4529)
Supplement: Supplementary file 1 — Supplementary Material [file HEC-31-1633-s001.docx]

Online Supplementary file

Correcting for discounting and loss aversion in composite time trade-off

Stefan A. Lipman*

Erasmus Centre for Health Economics Rotterdam, Erasmus School of Health Policy & Management, Erasmus University Rotterdam, [lipman@eshpm.eur.nl](mailto:lipman@eshpm.eur.nl), T: +31 10 408 2507

Arthur E. Attema

Erasmus Centre for Health Economics Rotterdam, Erasmus School of Health Policy & Management, Erasmus University Rotterdam, [attema@eshpm.eur.nl](mailto:attema@eshpm.eur.nl)

Matthijs M. Versteegh

Institute for Medical Technology Assessment, Erasmus University Rotterdam, [versteegh@imta.eur.nl](mailto:versteegh@imta.eur.nl)

Funding sources

This study was made possible through funding provided by the EuroQol Research Foundation (project number: 20190080R1).

Acknowledgements

The views expressed by the authors do not necessarily reflect the views of the EuroQol group. We also gratefully acknowledge the valuable assistance and comments provided by Elly Stolk and Benjamin Craig.

Conflicts of interest:

Matthijs Versteegh is a member of the EuroQol Group. All authors have received research grants from the EuroQol Research Foundation for work outside the scope of the submitted work.

# Contents

This Online Supplement contains a total of 4 appendices, as listed below.

- Apppendix A clarifies the differences between our approach and the general QALY model using a set of intuitive Figures.
- Appendix B lists the questions used for collecting demographics.
- Appendix C contains a set of screenshots and (translated) instructions used for each of the tasks in the experiment.
- Appendix D contains additional results on, inter alia, the differences between digital and personal composite time trade-off (cTTO) interviews and on isolated corrections.

# Appendix A – Our approach vs. general QALY model

In this appendix, we demonstrate and illustrate how the notation used for introducing reference-dependence into the QALY model, e.g. as outlined in Eq. 6, is equivalent to the general QALY model. We also show how the corrective approaches used (Eq. 8, 10 and 12) follow from this notation when loss aversion is introduced for losses in duration. For ease of interpretation, we include a set of figures that illustrates this equivalence and show how our notation applies for any reference-point (in the simple case where the reference-point is $\left( Q_{r},T_{r} \right)\boldsymbol{)}$, and leads to the corrective approach for ‘conventional’ TTO**.** Next, we provide substantiation for the extension to lead-time TTO in both Eq. 10 (constant alternative correction) and Eq. 12 (maximal BTD correction).

## A.1. Reference-dependent evaluation of $\left( \boldsymbol{Q}_{\boldsymbol{x}}\boldsymbol{,}\boldsymbol{T}_{\boldsymbol{x}} \right)$ and the general QALY model

Our model incorporates a reference-point$\left( Q_{r},T_{r} \right)$ into the general QALY model. Compared to that reference-point, profiles $\left( Q_{x},T_{x} \right)$ can entail gains and losses in both $Q$and $T.$Below we illustrate that Eq. 6 is equivalent to the general QALY model in all cases. Throughout, in the general QALY model $U\left( Q_{x},T_{x} \right)\boldsymbol{=}H\left( Q_{x} \right)L(T_{x})$.

### A.1.1. Gains/Losses in Q and T

Whenever $\left( Q_{x},T_{x} \right)$ consists of gains or losses in both dimensions with respect to $\left( Q_{r},T_{r} \right)$we have for gains: $Q_{x}≽Q_{r}$, $T_{x}$ $\geq$ $T_{r}$, and for losses: $Q_{x}\prec Q_{r}$, $T_{x}<T_{r}$. Figures A1 and A2 show that $H\left( Q_{x} \right)L\left( T_{x} \right)={H(Q}_{r})L\left( T_{r} \right)+\left( H\left( Q_{x} \right)-H\left( Q_{r} \right) \right)L\left( T_{x} \right)+$ $H\left( Q_{r} \right)\left( L\left( T_{x} \right)- L\left( T_{r} \right) \right)$. In other words, decomposing the total utility into the utility of the reference-point, a gain/loss part with respect to $Q_{r}$, and a gain/loss part with respect to $T_{r}$, respectively, does not affect the total utility.


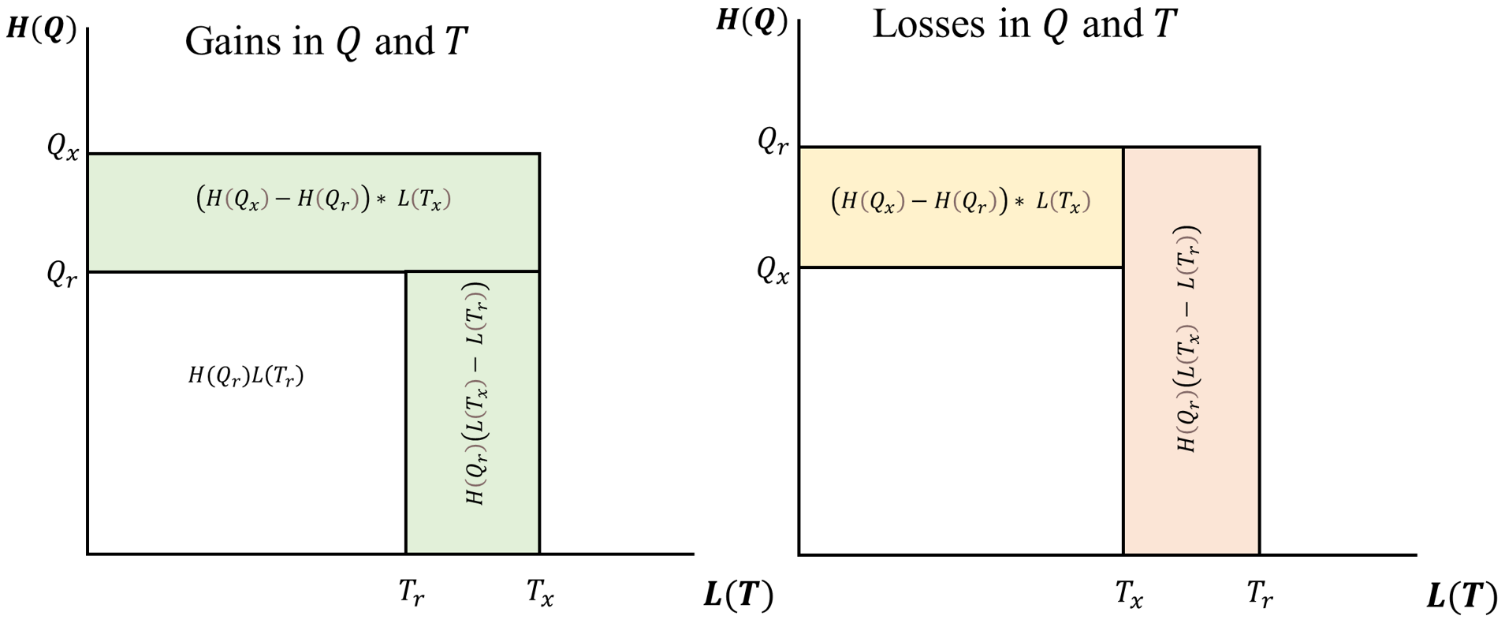


*Figure A1.* Gains in Q and T (in green) and Losses in Q (in orange) and T (in red) decomposed in our approach and their equivalence with the general QALY model*.* Note that the loss in T is multiplied by $\lambda$ whilst the loss in Q is not (hence the different colors).

### A1.2. Gain/Losses in Q or T

It is straightforward to see how this also holds for gains *or* losses in Q and T. In other words, whenever one of the following four holds:

i) $Q_{x}≽Q_{r}$, $T_{x}$ = $T_{r}$, (gains in Q only)

ii) ${Q_{x}\prec Q_{r}, T}_{x}=T_{r}$, (losses in Q only)

iii) ${Q_{x}\sim Q_{r}, T}_{x}\geq T_{r}$, (gains in T only), or

iv) ${Q_{x}\sim Q_{r}, T}_{x}<T_{r}$, (losses in T only).

In those cases, the total utility is decomposed in the utility of the reference-point and either a gain/loss part with respect to $Q_{r}$, or the gain/loss part with respect to $T_{r}$. As before, as can be seen from Figure A3 and A4, this does not affect the total utility which implies that $H\left( Q_{x} \right)L\left( T_{x} \right)={H(Q}_{r})L\left( T_{r} \right)+\left( H\left( Q_{x} \right)-H\left( Q_{r} \right) \right)L\left( T_{x} \right)+$ $H\left( Q_{r} \right)\left( L\left( T_{x} \right)- L\left( T_{r} \right) \right)$.


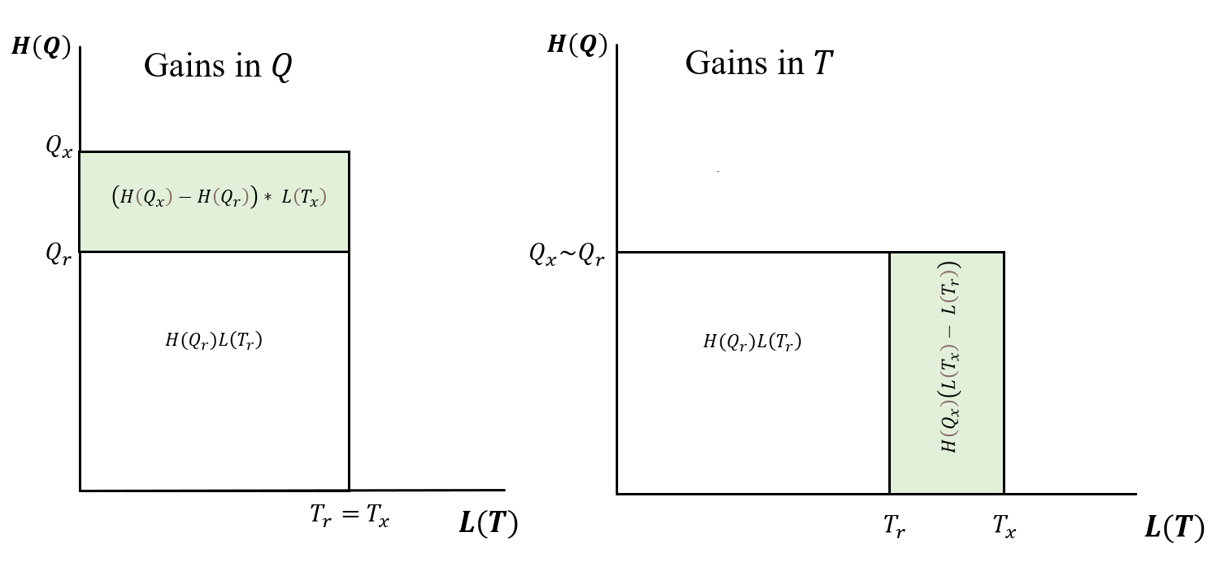


*Figure A3*. Gains in Q or T only decomposed in our approach and their equivalence with the general QALY model.


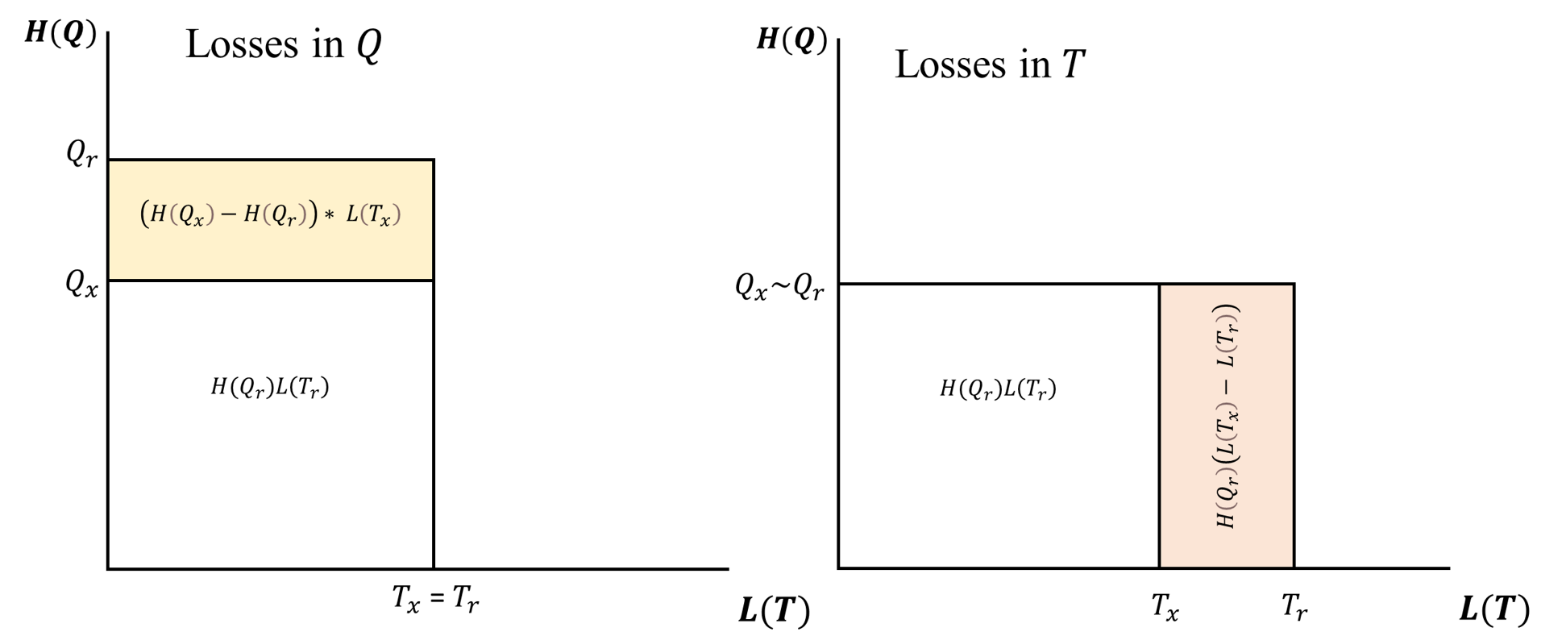


*Figure A4*. Losses in Q or T only decomposed in our approach and their equivalence with the general QALY model.

### A.1.3. Gains in Q and losses in T (as assumed in conventional TTO)

In the corrective approach applied to conventional TTO it is typically assumed that the time in impaired health is considered to be the reference-point. As outlined in the main text, this implies that a gain in Q is traded off against a loss in T, i.e.: we have $\left( Q_{x},T_{x} \right)$ with $\left( Q_{r},T_{r} \right)$ and $Q_{x}≽Q_{r}$, $T_{x}\leq T_{r}.$ Our approach is illustrated in Figure A4.


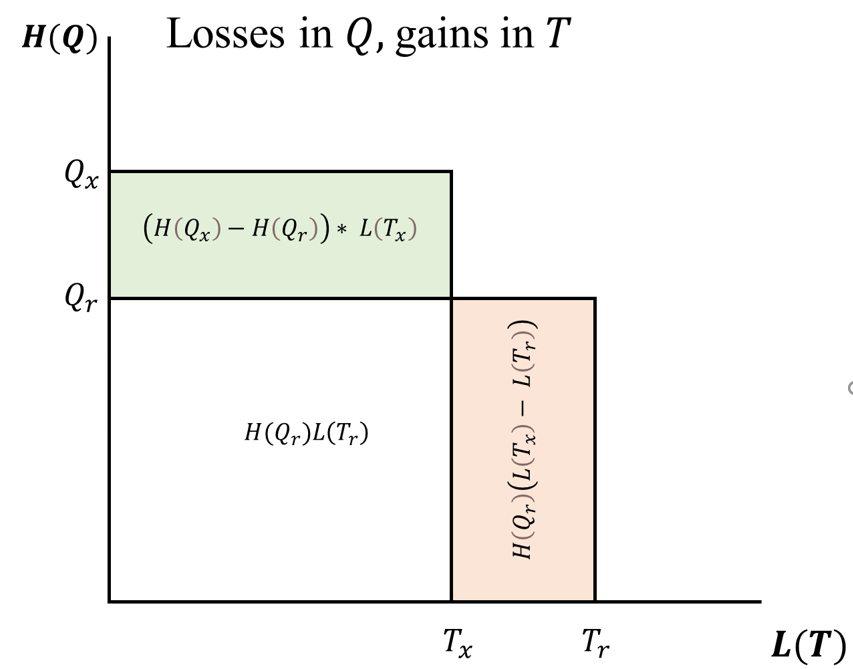


Figure A5. Gains in Q (green) and losses in T (red) decomposed in our approach and their equivalence with the general QALY model. Note that the loss in T is multiplied by $\lambda$.

### A.1.4. From Eq. 6 to Eq. 8

Standard TTO involves indifferences of the form $\left( FH,T \right)\sim\left( Q_{r},T_{r} \right).$In our decomposition, this implies: $U\left( FH,T \right)=U\left( Q_{r},T_{r} \right)$, i.e.

${H(Q}_{r})L\left( T_{r} \right)+\left( H\left( FH \right)-H\left( Q_{r} \right) \right)L\left( T \right)+$ $H\left( Q_{r} \right)\left( L\left( T \right)- L\left( T_{r} \right) \right)={H(Q}_{r})L\left( T_{r} \right)+\left( H\left( Q_{r} \right)-H\left( Q_{r} \right) \right)L\left( T_{x} \right)+$ $H\left( Q_{r} \right)\left( L\left( T_{r} \right)- L\left( T_{r} \right) \right)$. Simplifying and incorporating that $H\left( FH \right)=1$and that we take $T_{r}=10,$we get:

${H(Q}_{r})L\left( 10 \right)+\left( 1-H\left( Q_{r} \right) \right)L\left( T \right)+$ $H\left( Q_{r} \right)\left( L\left( T \right)- L\left( 10 \right) \right)={H(Q}_{r})L\left( 10 \right)$.

It may be useful to note that this evaluation is identical to the derivation of $\left( FH,T \right)\sim\left( Q_{r},T_{r} \right)$ in the general QALY model. Only when we assign $\lambda$to the decomposed loss in life duration we deviate from the general QALY model, and we get:

${H(Q}_{r})L\left( 10 \right)+\left( 1-H\left( Q_{r} \right) \right)L\left( T \right)+$ $H\left( Q_{r} \right)\lambda\left( L\left( T \right)- L\left( 10 \right) \right)={H(Q}_{r})L\left( 10 \right)$.

Rearranging and simplifying gives Eq. 8.

## A.2. An extension to lead-time TTO

The extension of this approach to lead-time TTO follows from the notation and decomposition. For the sake of brevity, we directly consider the cases assumed for constant alternative and maximal BTD correction, respectively (rather than providing proof of equivalence to the general QALY model in nearly all cases).

### A.2.1. Correction based on constant alternative

When we apply constant alternative correction, we assume that $\left( FH,10; Q,11:20 \right)$ is the reference-point. Applying the same logic as captured in Eq. 6 requires introducing some additional notation. We will write $\left( Q_{ra},T_{ra};Q_{rb},T_{ra}+1:T_{rb} \right)$ to denote the reference-point that consists of a health profile in which quality of life is equal to$Q_{ra}$ in $\mathrm{periods} 1, 2, \ldots\mathrm{to} T_{ra},$ followed by a health profile with $Q_{rb}$for period $T_{ra}+1, \ldots, T_{rb}$, with indexes a and b being added to distinguish between the two reference profiles. Indifferences in lead-time TTO, when applying constant alternative correction, therefore, can be denoted as: ${(Q}_{LT},T_{LT})\sim\left( Q_{x},T_{x};Q_{y},T_{x}+1:T_{y} \right)$ with $\left( Q_{ra},T_{ra};Q_{rb},T_{ra}+1:T_{rb} \right)$, and ${Q_{LT}\sim Q}_{x}\sim Q_{ra}\succ Q_{y}\sim Q_{rb}$, ${{T_{LT}<T}_{x}=T}_{ra}<T_{y}=T_{rb}.$The LT subscript is introduced here to avoid confusion, and captures the elicited full health equivalent in lead-time TTO. In the general QALY model, such indifferences are represented as: $U{(Q}_{LT},T_{LT})=U\left( Q_{x},T_{x};Q_{y},T_{x}+1:T_{y} \right),$ i.e. $H{(Q}_{LT})L\left( T_{LT} \right)={H(Q}_{x})L\left( T_{x} \right)$+ ${H(Q}_{y})\left( L\left( T_{y} \right)-L\left( T_{x} \right) \right)$.

Our approach is similar, we decompose the outcomes in the utility of the reference-point and gains/losses in T and Q. Note, however, that in this case, that decomposition is applied to reflect that the reference-point consists of two profiles. That is:

$U\left( {(Q}_{LT},T_{LT} \right)={H(Q}_{ra})L\left( T_{ra} \right)+{H(Q}_{rb})\left( L\left( T_{rb} \right)-L\left( T_{ra} \right) \right)+{H(Q}_{LT})(L\left( T_{LT} \right)-L(T_{ra})+$ ${H(Q}_{rb})\left( L\left( T_{ra} \right)-L\left( T_{rb} \right) \right)$ and

$U\left( Q_{x},T_{x};Q_{y},T_{x}+1:T_{y} \right)$= ${H(Q}_{ra})L\left( T_{ra} \right)+{H(Q}_{rb})\left( L\left( T_{rb} \right)-L\left( T_{ra} \right) \right)$.

As can be seen from Figure A5, this evaluation is identical to the evaluation of the same indifference under the general QALY model (without reference-dependence). We obtain Eq. 9, and thus Eq. 10, by including $\lambda$ for losses in T, rearranging and realizing that ${Q_{LT}=Q}_{x}=Q_{ra}=FH,$and ${{T_{LT}<T}_{x}=T}_{ra}=10<T_{y}=T_{rb}=20.$


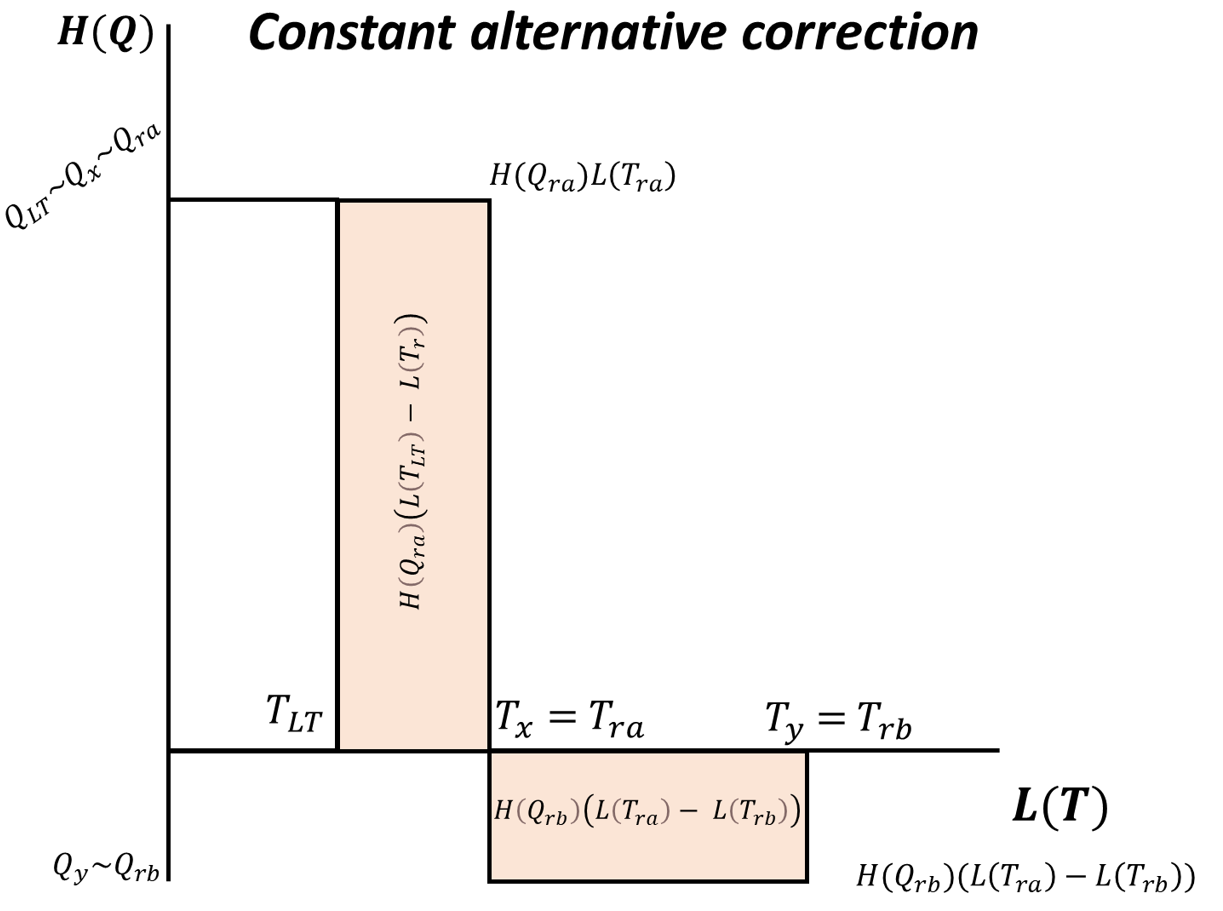


*Figure A6.* Constant alternative correction decomposed in our approach and equivalence with the general QALY model. Note that losses in T are multiplied by $\lambda$.

### A.2.2. Correction based on maximal BTD time

When we apply maximal BTD correction we assume that $\left( FH,10 \right)$ is the reference-point. In other words, lead-time TTO involves eliciting ${(Q}_{LT},T_{LT})\sim\left( Q_{x},T_{x};Q_{y},T_{x}+1:T_{y} \right)$ with $\left( Q_{r},T_{r} \right)$ and $Q_{x}\sim Q_{r}\succ Q_{y}$ and ${{T_{LT}<T}_{x}=T}_{r}<T_{y}$. As a reminder, under the general QALY model this indifference is represented as: $H{(Q}_{LT})L\left( T_{LT} \right)={H(Q}_{x})L\left( T_{x} \right)$ + ${H(Q}_{y})\left( L\left( T_{y} \right)-L\left( T_{x} \right) \right)$. In our approach$, {(Q}_{LT},T_{LT})$ is decomposed into the loss in lifetime and gain in Q, i.e. as discussed in section A.1.2. (for standard TTO with losses in T). That is, realizing that there is no gain/loss in Q to decompose, we have:
$U{\left( Q_{LT},T_{LT} \right)=H(Q}_{r})L\left( T_{r} \right)+H\left( Q_{r} \right)\left( L\left( T_{LT} \right)- L\left( T_{r} \right) \right)$*.*

Applying the same approach as in Eq. 6 to $\left( Q_{x},T_{x};Q_{y},T_{x}+1:T_{y} \right)$ with $\left( Q_{r},T_{r} \right)$yields:

$U\left( Q_{x},T_{x};Q_{y},T_{x}+1:T_{y} \right)=$ ${H(Q}_{r})L\left( T_{r} \right)+H\left( Q_{y} \right)\left( L\left( T_{y} \right)- L\left( T_{r} \right) \right)$.

Figure A7 shows that these decompositions (before adding $\lambda$ for losses in T for${(Q}_{LT},T_{LT})$ are equivalent to the evaluation in the general QALY model. We obtain Eq. 11, and thus Eq. 12, by incorporating $\lambda$ for losses in T, rearranging and realizing that $Q_{x}=Q_{r}=FH$ and ${{T_{LT}<T}_{x}=T}_{r}=10<T_{y}=20$.


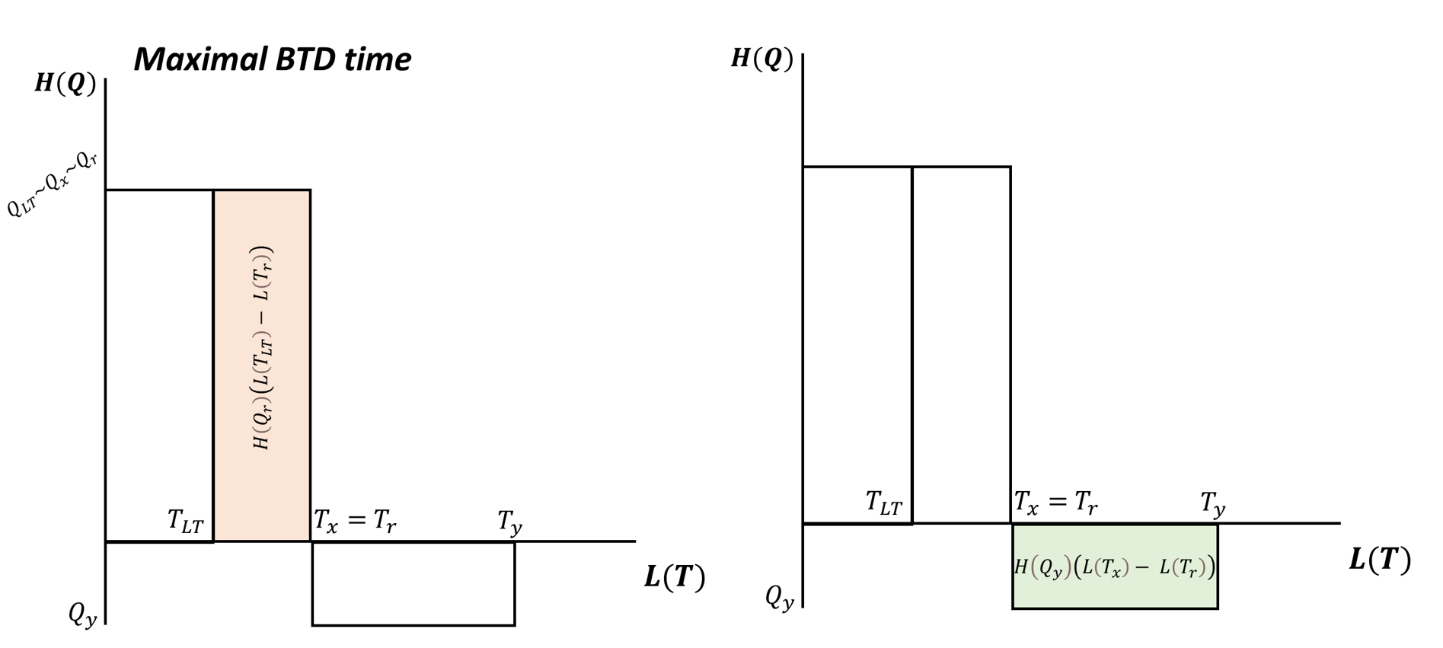


*Figure A7.* Maximal BTD correction decomposed in our approach, with evaluation of $\left( Q_{LT},T_{LT} \right)$ on the left (with $\lambda$ added to losses in T) and evaluation of $\left( Q_{x},T_{x};Q_{y},T_{x}+1:T_{y} \right)$ on the right.

# Appendix B – Questions used for measuring demographics

1. (sex) What is your sex? (male; female; other)
2. (age) What is your age (in years)?
3. (household income) What is your yearly income? (less than 15.000 euro; 15.000-30.000 euro; 30.000-60.000 euro; more than 60.000 euro)
4. (SLE) How old do you think you will become?
5. What is the maximum age you would want to become?
6. Are you religious? (Yes, my religion is: …; No)
7. Do you believe there is such a thing as life after death (Yes, I do believe that; I’m not sure; No, I don’t believe that)
8. What do you think about euthanasia? (I feel that euthanasia should not be allowed under any circumstances; I feel that euthanasia should be allowed under strict circumstances (such as in case of unbearable suffering with no hope for improvement); I feel that euthanasia should be allowed, but only after careful consideration and professional counselling; I feel that anyone should be able to choose for euthanasia freely).

# Appendix C – Screenshots and instructions used for the experiment

## C.1. Screenshots for cTTO interview

Figure C1 shows an example of the software used to operationalize TTO (the EQ-PVT software). All labels and information was presented in Dutch.
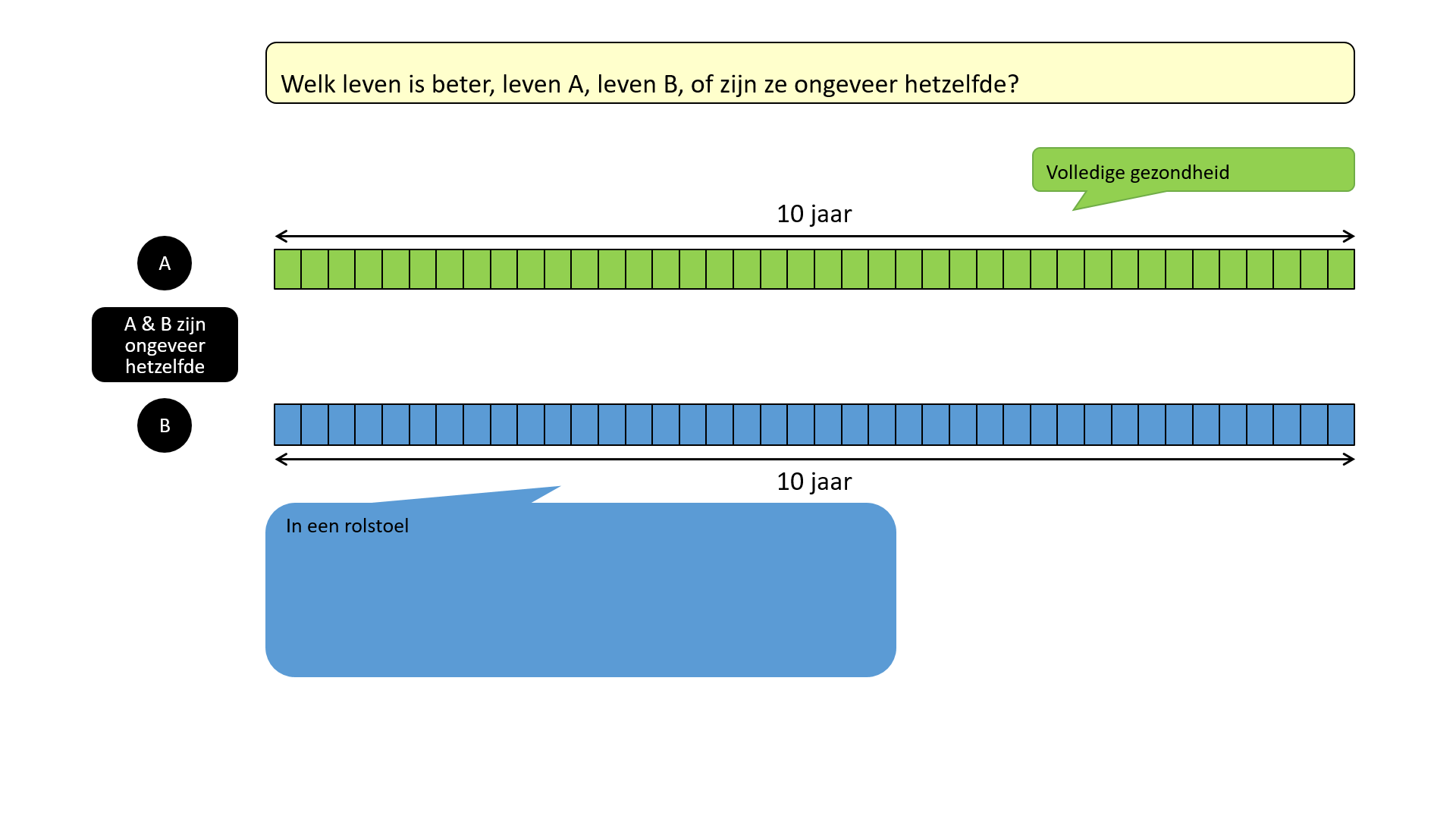


*Figure C1.* EQ-PVT interface for TTO wheelchair example

When respondents faced the lead-time TTO version, they saw the interface in Figure C2.


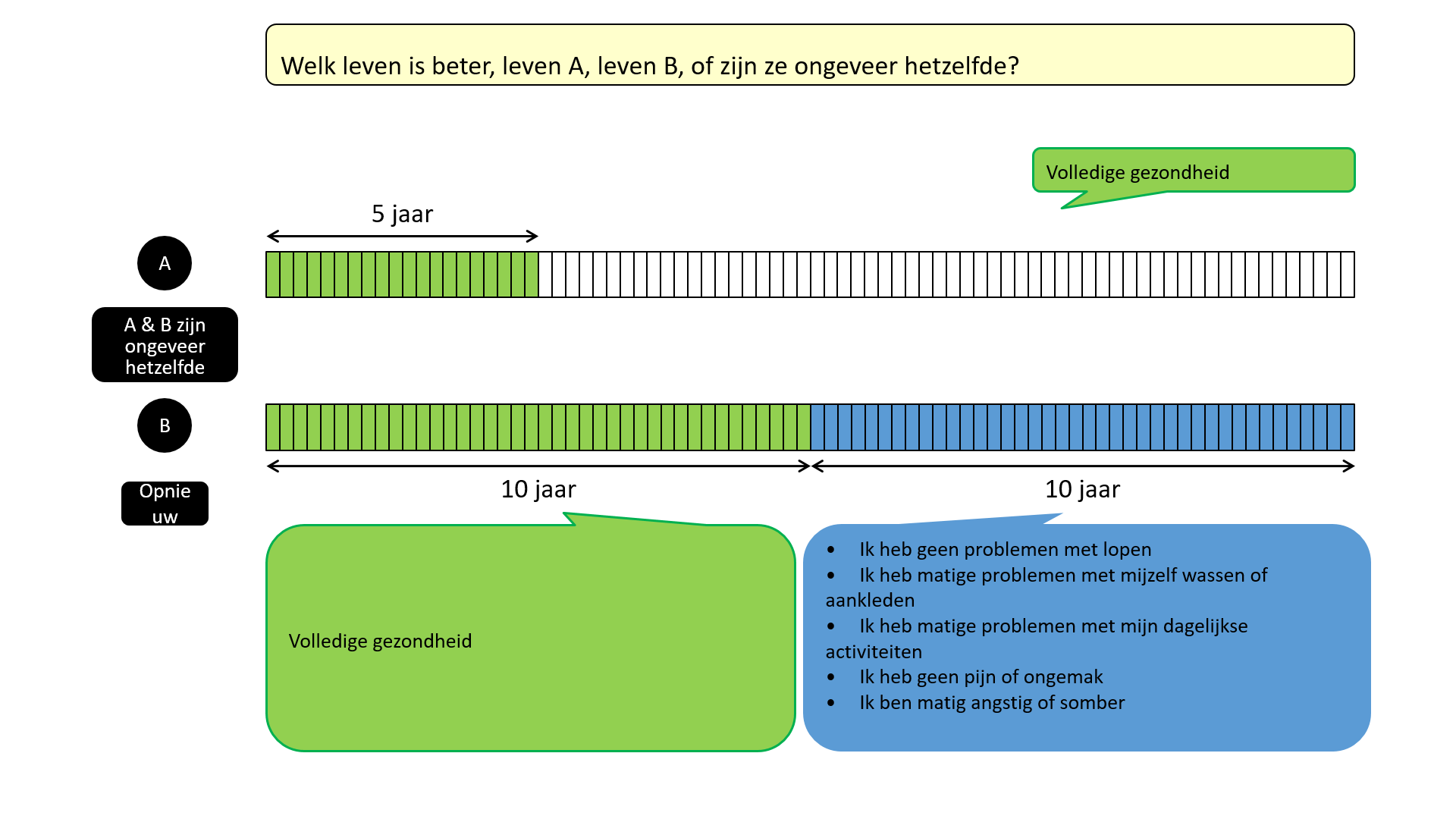


*Figure C2.* EQ-PVT interface for lead-time TTO

## C.2. Elicitation of loss aversion

Figure C3 shows the interface used for the non-parametric method used for measuring loss aversion. The task used to measure loss aversion was introduced with instruction (translated from Dutch) found in Box C1.

**Box C1. Instructions used for elicitation of loss aversion (10 years as reference-point)**

Imagine you will live for 10 more years in full health. After this period, you will pass away directly and without pain. For the next part of the experiment you will get to choose between various options that might alter your lifespan. Such changes might either extend your lifespan beyond 10 years, or shorten your lifespan. For example, you can choose a treatment that has the possibility to lengthen your lifespan with 5 years (on top of the 10 years you still had to live). Not all treatments have a sure outcome, sometimes there is a risk involved. For example, a treatment might have a chance of 50% to shorten your lifespan with 5 years (relative to the 10 years you still had to live). In the next part you will be asked several times to choose between such treatments, again with the purpose to find the moment you think both treatments are equally good.


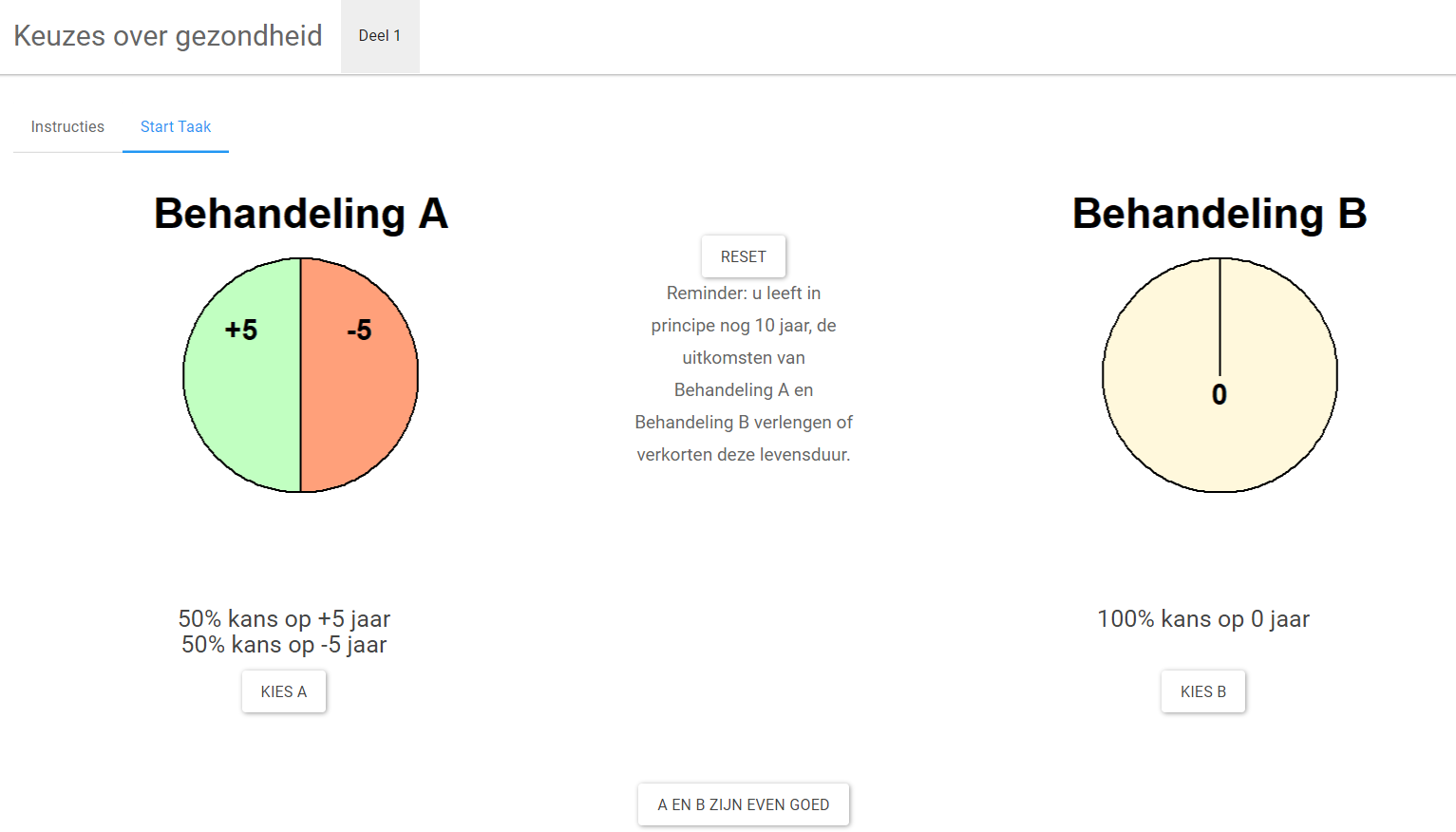


*Figure C3.* Interface used for the non-parametric method for measuring loss aversion

## C.3. Elicitation of discounting

Figure C4 shows the interface used for the direct method for measuring discounting. The instruction used (translated from Dutch) can be found in Box C2.

**Box C2. Instructions used for elicitation of discounting**

Imagine your current health is as described below:

*Chronic back pain*

Slight problems walking about.

No problems washing or dressing myself.

No problems with daily activities.

Slight pain or discomfort.

Not anxious or depressed.

There is an option to go through a treatment that will help you return to a perfect health state for a certain period of time, meaning a health state in which you are able to perform all activities without any problems and in which you have no health complaints, even with higher age. Unfortunately, this treatment does not have a lasting effect.

After a certain period of time the problems will return and you will go back to the health state as described under chronic back pain. You can choose between two possible treatments. The first treatment’s effect will occur sooner than the effect of the second treatment. The duration of the relief of the symptoms may differ between the two treatments. In all other aspects, both treatments are equal. You expected lifespan will also be equal for both treatments. So the question is: would you rather have the improved health state sooner or later, and when exactly?

For a number of different periods in perfect health, you will now be asked to choose between these two treatments. Please note that all numbers can change. After several of these choices follows the opportunity to alter the treatments such that both treatments are approximately equal.


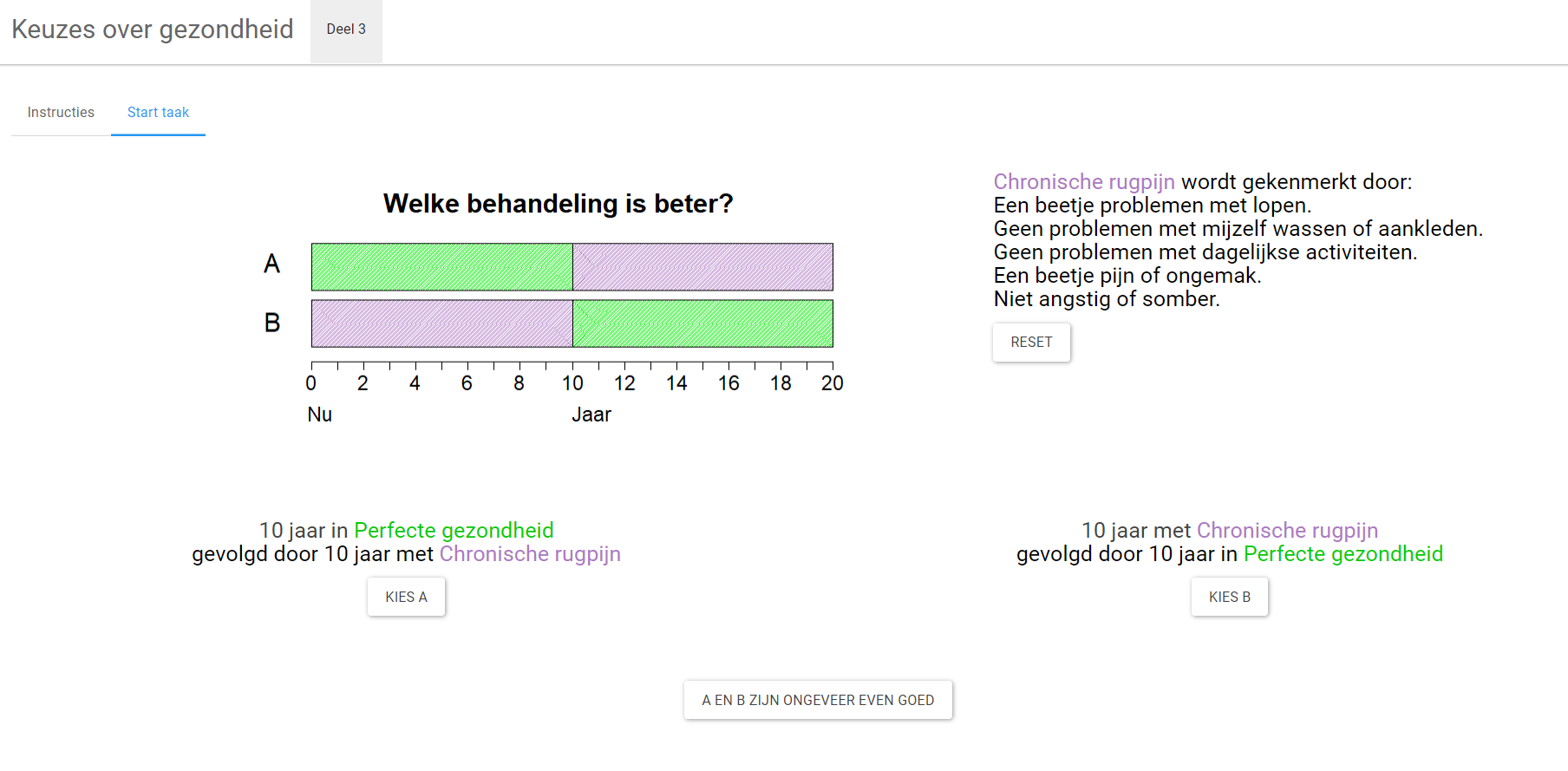


*Figure C4.* Interface used for the direct method for measuring discounting

## C.4. Validation task

Figure C5 shows the interface used for the validation task. The instruction used (translated from Dutch) can be found in Box C3.

**Box C3. Instructions used for validation task**

Health is often described as a combination of life expectancy and health status e.g. quality of life. In the first part of this interview you have completed several choice tasks with the purpose of finding the value of life with a reduced quality (for various conditions). This information is relevant because it can be used to express in numbers how much health can be won with newly developed medicines. Quality of life is often expressed on a scale from 0 to 1, with the value of 0 meaning ‘death’ and the value of 1 meaning the best possible health state (perfect health). Conditions worse than death get a value below 0 (negative value). Health conditions like chronic back pain will fall somewhere in between these values, and people can differ in how good or bad they think chronic back pain is compared to perfect health.

This subjective scale from 0 to 1 is often used because it enables us to compute ‘Quality-adjusted life years (QALYs)’. This outcome measure, in which length and quality of life are combined, can be computed by multiplying life years with the numeric value that is given to the quality of life (i.e.: the value of chronic backpain on a scale of 0 to 1).

For example: many people say they are perfectly healthy. As long as they can stay in perfect health every year, every one of these years counts as 1 QALY (1 year multiplied by a value of 1). But now imagine someone having rheumatoid arthritis, which leads to this person having a lot of trouble walking and being in a lot of pain. The life years of this person will be of lower quality, therefore being worth less than 1 QALY a year. If, for example the quality of life of a person with rheumatoid arthritis decreases with 50%, every remaining life year of will be worth ½ QALY.

In the first part of this interview you have completed several choice tasks in which you imagined living in conditions described by 5 dimensions. These kind of choice tasks, in which people like you (probably in reasonably to very good health) imagine living in such conditions and make choices about health, allow us to find the value these people assign to these health conditions on a scale of 0 to 1 (the QALY-scale). If your choices showed there were health conditions you thought worse than death, these conditions have a negative value. In this part of the interview we will show you what your choices from the first part of the interview suggest about the value you assigned to life in these health states on a scale of 0 to 1 (from death to perfect health). However, your choices only give us rough estimate, so in the final part of this interview we will ask you to help us find the exact value of this health state you encountered before.

In this part of the interview we will show you our best estimate of what we think is the value you assign to every health state on the QALY-scale. We will ask you whether the value we estimated is right, or if maybe this value should be higher or lower. If necessary you can adjust these values to have them better resemble what you think is the value of living in these health conditions.


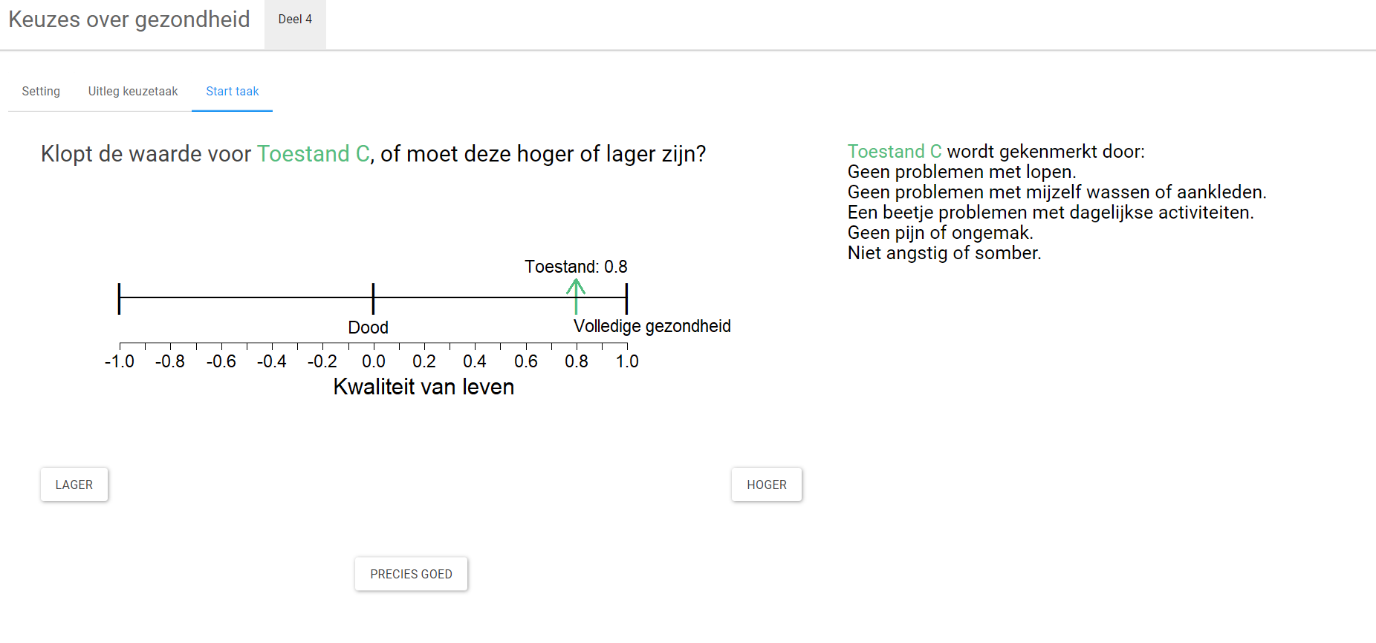


*Figure C5*. Interface used for validation task

# Appendix D– Additional analysis.

## D.1. Comparing digital and personal cTTO interviews

Figure D1 and Figure D2 show the distribution of elicited cTTO utilities for interviews completed in person or digitally. The distribution appears similar. Indeed, no differences were observed for all health states for elicited, confirmed or corrected (for both specifications) cTTO utilities (t-tests, all p’s > 0.05), with a single exception: the confirmed cTTO utility for health state 24443 (Wilcoxon test, p’s = 0.04). Correcting for multiple hypothesis testing with Bonferroni correction rendered these two exceptions non-significantly different (adjusted p = 0.24). To determine of the data quality differed between personal and digital interviews, we also adapted the approach reported in Alava et al. (2020) and find no differences in data quality (see Table D1). Furthermore, we find no differences between digital and personal interviews for the EQ-5D-5L (and EQ-VAS), the loss aversion coefficients elicited, the elicited durations in the direct method ($T_{d1/8}, T_{d1/4},T_{d1/2},T_{d3/4}$ and $T_{d7/8}$), or the AUC (Wilcoxon test, all p’s > 0.15).

Hence, the only relevant difference between personal and digital TTO interviews appears to be the age of the recruited sample (see Table 1 in main text).

## D.2. Isolated corrections (i.e. only for loss aversion or discounting)

Below we reprint Figure 1 for isolated corrections. That is, Figure D3 shows cTTO utilities when only loss aversion is corrected and Figure D4 shows the same utilities when only discounting is corrected for. As such, the corrected cTTO utilities Figure D3 are derived by applying Eq. 8, 10, and 12 with the assumption that $L\left( T \right)$ is linear (we still used the scaling convention used in our model). The corrected utilities in Figure D4 are derived by applying Eq. 8, 10 and 12 assuming $\lambda=1.$ Comparing Figure D3 and D4 shows that the corrected cTTO utilities are lower than elicited cTTO utilities as a result of correction for loss aversion. This is especially true when comparing constant correction (no loss aversion for lead-time TTO) and maximum BTD correction (loss aversion for lead-time TTO included). Also, comparing the two figures shows that without loss aversion, Eq. 10 and 12 yield exactly the same results.

**Table D1.** Data quality in personal TTO and digital TTO interviews, with quality indicators adapted from (Alava et al., 2020)

|  | **Personal TTO (n=36)** | | **Digital TTO**  **(n=113)** | | $\chi^{2}$ | *p* |
| --- | --- | --- | --- | --- | --- | --- |
| **Problematic responder types** | N | % | N | % |  |  |
| All 6 health states same utility | 0 | 0 | 0 | 0 | - | - |
| One or more health state(s) ranked the same as 55555 | 8 | 22.2 | 32 | 28.3 | 0.25 | 0.62 |
| One or more health state(s) ranked strictly lower than 55555 | 3 | 8.3 | 3 | 2.6 | 1.07 | 0.30 |
| 11211 same utility as 55555* | 0 | 0 | 0 | 0 | - | - |
| Fewer than 4 distinct values | 1 | 2.8 | 7 | 6.1 | 0.13 | 0.72 |
| Utility of -1, -0.5, 0, 0.5 or 1 for all 6 health states | 0 | 0 | 0 | 0 | 0 | - |
| No negative utilities | 6 | 16.7 | 13 | 11.4 | 0.29 | 0.59 |
| No use of half year increments in TTO | 7 | 19.4 | 31 | 27.4 | 0.55 | 0.46 |
|  | **Personal TTO (n=216)^a^** | | **Tele-TTO**  **(n=678)^a^** | | $\chi^{2}$ | *p* |
| **All responses compiled** | N | % | N | % |  |  |
| Non-trading (utility of 1) | 36 | 16.7 | 98 | 14.4 | 0.47 | 0.49 |
| All-in trading (utility of -1) | 23 | 10.6 | 95 | 14.0 | 1.34 | 0.25 |
|  | **Personal TTO (n=360)^b^** | | **Tele-TTO**  **(n=1130)^b^** | | $\chi^{2}$ | |
| **All potential violations** | N | % | N | % |  |  |
| Strict violations of logical consistency  (e.g. utility of 13313 > 24443) | 8 | 2.2 | 6 | 0.05 | 6.71 | **0.01** |
| Weak violations of logical consistency  (e.g. utility of 13313 >= 24443) | 27 | 7.5 | 89 | 7.8 | 0.02 | 0.90 |

**Note:** the sample size for the observation with ^a^ is larger as each respondent has 6 potential observations. Sample sizes with superscript ^b^ are even larger as each respondent had 10 potential violations of logical consistency.


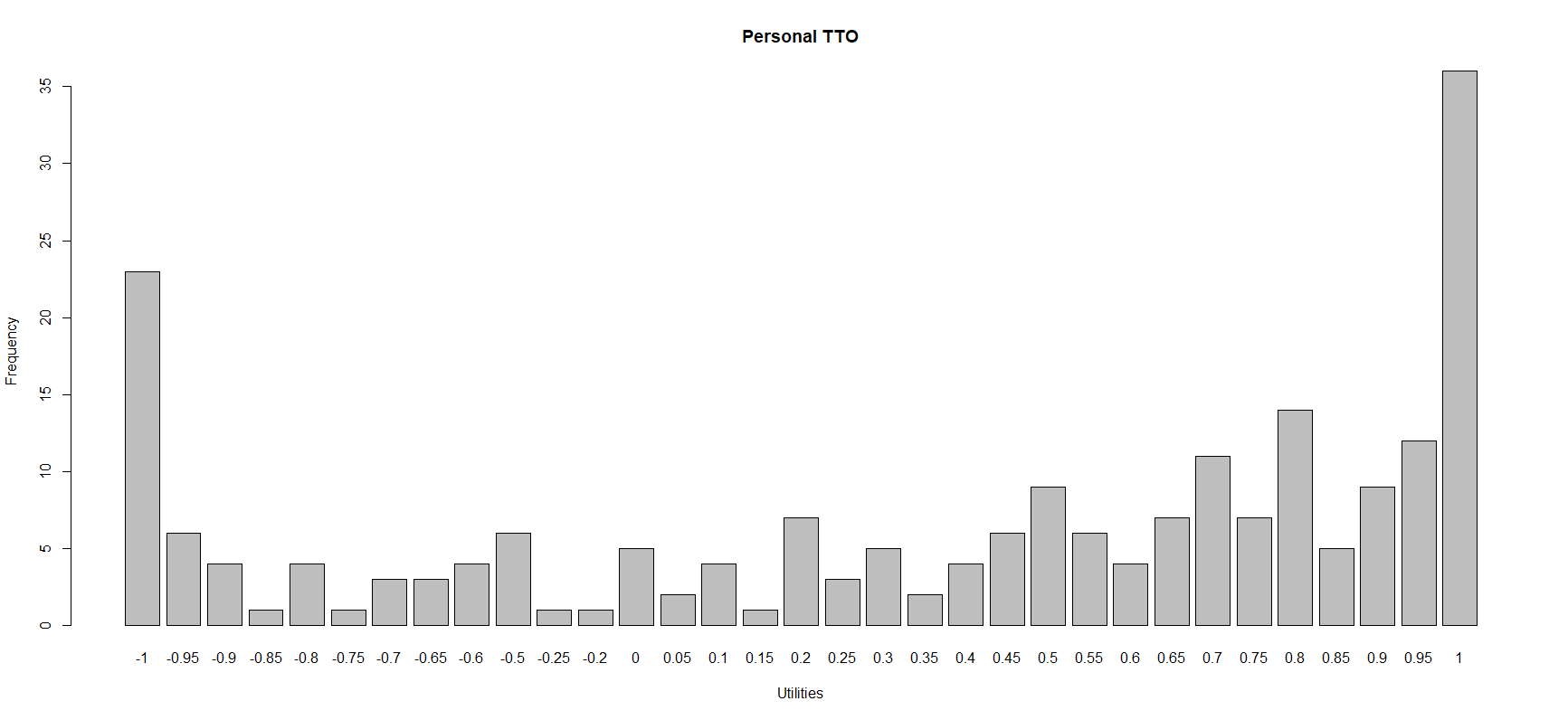


*Figure D1*. Elicited personal TTO utilities in personal TTO (*n* =36 observations for 6 health states)


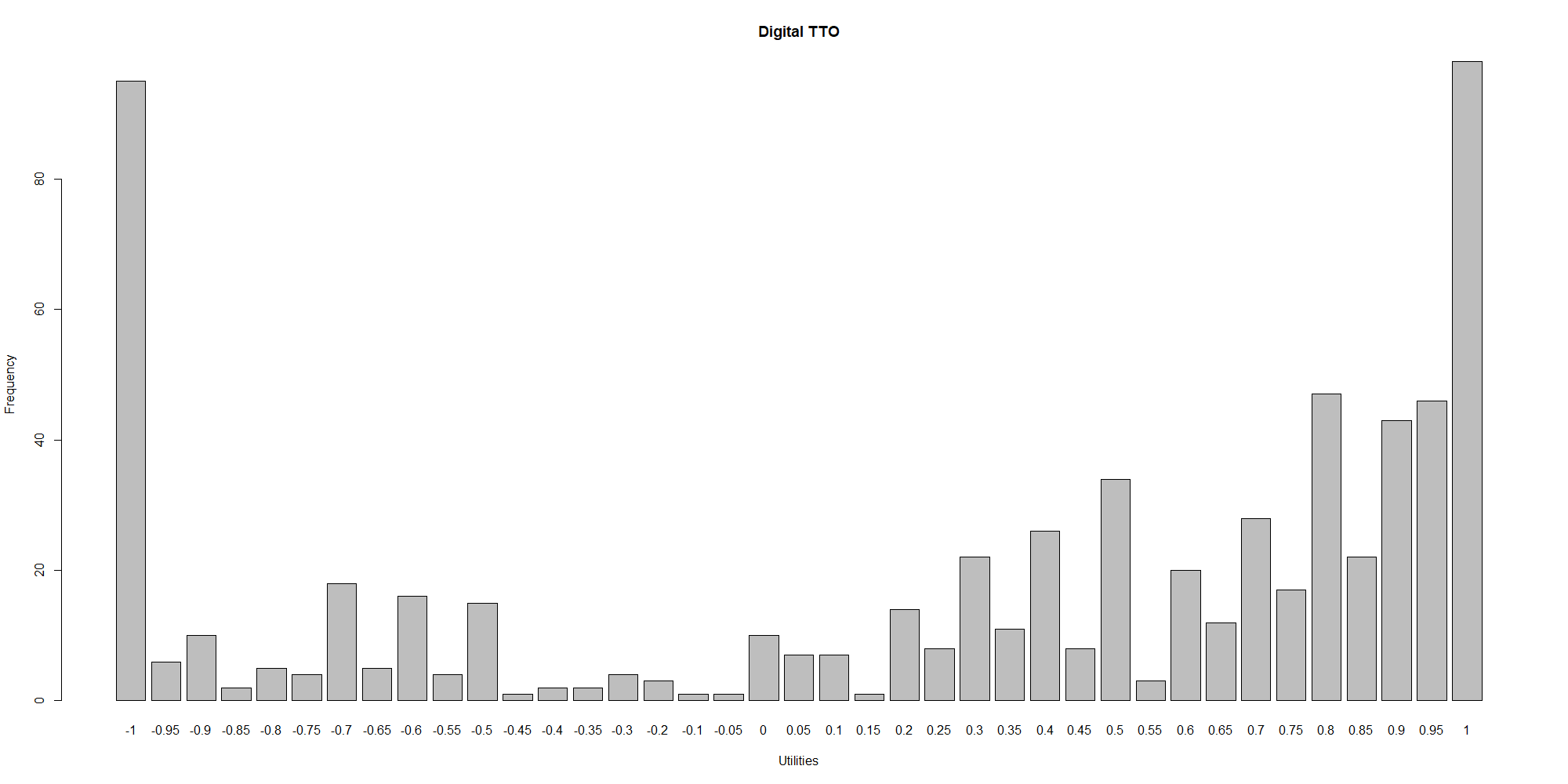
*Figure D2*. Elicited TTO utilities digital TTO interviews (*n* =113 observations for 6 health states)


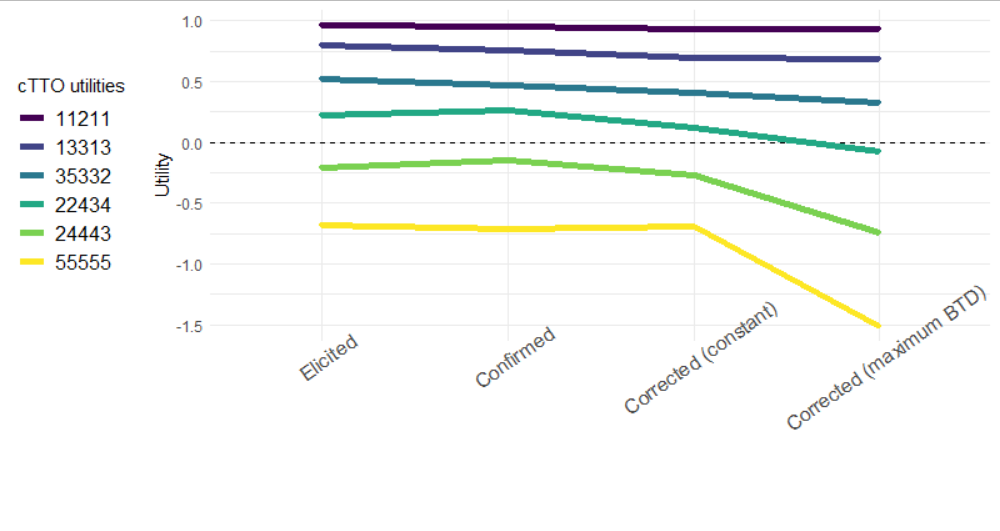


*Figure D3*. cTTO utilities for isolated corrections, i.e. when only correcting for loss aversion.


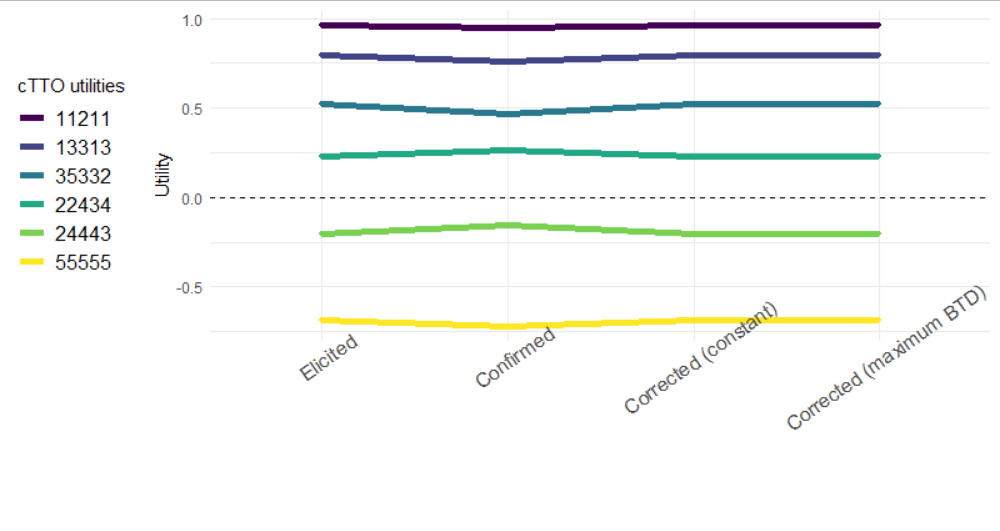


*Figure D4.* cTTO utilities for isolated corrections, i.e. when only correcting for discounting.
